# Synaptotagmin 1 clamps synaptic vesicle fusion in mammalian neurons independent of complexin

**DOI:** 10.1038/s41467-019-12015-w

**Published:** 2019-09-09

**Authors:** Nicholas A. Courtney, Huan Bao, Joseph S. Briguglio, Edwin R. Chapman

**Affiliations:** Department of Neuroscience and Howard Hughes Medical Institute, University of Wisconsin-Madison, 1111 Highland Ave., Madison, WI 53705 USA

**Keywords:** Neuroscience, Synaptic transmission, Synaptic vesicle exocytosis

## Abstract

Synaptic vesicle (SV) exocytosis is mediated by SNARE proteins. Reconstituted SNAREs are constitutively active, so a major focus has been to identify fusion clamps that regulate their activity in synapses: the primary candidates are synaptotagmin (syt) 1 and complexin I/II. Syt1 is a Ca^2+^ sensor for SV release that binds Ca^2+^ via tandem C2-domains, C2A and C2B. Here, we first determined whether these C2-domains execute distinct functions. Remarkably, the C2B domain profoundly clamped all forms of SV fusion, despite synchronizing residual evoked release and rescuing the readily-releasable pool. Release was strongly enhanced by an adjacent C2A domain, and by the concurrent binding of complexin to *trans*-SNARE complexes. Knockdown of complexin had no impact on C2B-mediated clamping of fusion. We postulate that the C2B domain of syt1, independent of complexin, is the molecular clamp that arrests SVs prior to Ca^2+^-triggered fusion.

## Abstract (web summary)

The molecular identity of the clamp that arrests the fusion machinery such that synaptic vesicles are docked and primed to release neurotransmitters remains controversial. In this study, the authors use truncation mutants of synaptotagmin (syt) 1 and animal models to demonstrate that the C2B domain of syt1, and not complexin, is solely responsible for the reduction of the spontaneous release at the presynapse

## Introduction

Neurons communicate though precisely controlled release of neurotransmitters from presynaptic terminals. During an action potential, Ca^2+^ influx through voltage-gated Ca^2+^ channels triggers rapid fusion of neurotransmitter-containing synaptic vesicles (SVs) with the plasma membrane. This fusion step is catalyzed by a complex of three SNARE proteins^1^: a vesicular SNARE (v-SNARE) on SVs and a heterodimer of target membrane SNAREs (t-SNAREs) on the plasma membrane. Progressive zippering of v- and t-SNAREs, into four-helix bundles, is thought to supply the force needed to merge membranes^2^. Unlike SV exocytosis, fusion mediated by reconstituted SNAREs is constitutive and is unaffected by Ca^2+^^3^. Synaptotagmin (syt) 1, a transmembrane SV protein that senses Ca^2+^ via tandem C2-domains termed C2A and C2B, imparts Ca^2+^-sensitivity to SNARE-mediated fusion in reconstituted systems^4^. In neurons, syt1 couples Ca^2+^ influx to synchronous SV release^5,6^. However, the molecular identity of the fusion clamp that prevents constitutive SNARE-mediated fusion in nerve terminals^7^, before the action of Ca^2+^ and syt1, is controversial and remains a topic of great interest. Two classes of proteins have emerged as potential fusion clamps: syt1 itself and complexin I/II.

Genetic disruption of syt1 abolishes synchronous neurotransmitter release^5,6^. Furthermore, syt1 may inhibit fusion in the absence of action potential induced Ca^2+^ entry. In the absence of Ca^2+^, the cytoplasmic domain of syt1 slows the rate of SNARE-mediated fusion in a reconstituted system^4,8^, and, in neurons, loss of syt1 increases the frequency of spontaneous neurotransmitter release events^8,9^. However, increased spontaneous fusion was not observed in all syt1 KO preparations^5^, and it is unclear whether this apparent arresting function arises from intrinsic properties of syt1^10,11^, or instead emerges as a neuronal network property^12,13^. Thus, whether and how syt1 clamps SVs remains an open question^14^.

Complexin I/II, the other putative fusion clamps, are small soluble proteins that bind assembled SNARE complexes^15^. Loss of complexin impairs evoked SV fusion^15^; however, whether loss of complexin in mammalian neurons affects the frequency of spontaneous release, which indicates a clamping function, remains highly debated. One group reported an increase in the rate of spontaneous events when complexin I/II were knocked-down, and concluded the complexin was a fusion clamp^16–20^. The underlying model postulates that complexin prevents full zipping of the SNARE complex through repulsive interactions with SNAREs and/or membranes^21,22^. Then, during synchronous release, Ca^2+^•syt1 would bind the SNARE complex and displace complexin to trigger fusion^23^. However, complexin and syt1 have been shown to concurrently bind SNAREs^20,24^, and Ca^2+^•syt1 does not appear to displace complexin from SNARE complexes^24^. Furthermore, the majority of studies agree that in complexin KO neurons, there is either no change or a modest decrease in the rate of spontaneous events^22,25–30^. These latter studies suggest that mammalian complexin promotes, rather than clamps, fusion.

Here, we examined whether the individual C2-domains of syt1 execute discrete functions when regulating exocytosis. Syt1 deletion constructs lacking either the C2A or C2B domain were expressed and characterized in cortical neurons cultured from syt1 KO mice. We found that the individual C2 domains exert specific, distinct effects on synaptic transmission. The most striking finding was that the C2B domain of syt1 was an extremely potent clamp that inhibited all forms of SV fusion. This clamping activity was not secondary to defects in the docked and primed pool of SVs; C2B was both necessary and sufficient to fully rescue the readily releasable pool (RRP) in syt1 KO neurons^12,31^. Instead, we postulate that direct, Ca^2+^-independent interactions between the bottom of C2B (directly opposite the Ca^2+^-binding loops) and the *trans*-SNARE complex underlie this potent clamping activity. Surprisingly, KD of complexin I/II had no effect on the ability of syt1 C2B to clamp fusion. Instead, our results indicate that complexin promotes Ca^2+^•syt1 regulated fusion, both in in vitro fusion assays and in neurons. The C2A domain plays a similar role as complexin, but more profoundly increases release probability. We conclude that the C2B domain of syt1, independent of complexin, is the molecular clamp that arrests SVs in a fusogenic state. Following Ca^2+^-entry, syt1 and complexin, while being bound to the same *trans*-SNARE complex, act in concert to trigger rapid, efficient, and evoked fusion.

## Results

### Either C2 domain is sufficient to target syt1 to synaptic vesicles

To determine whether the individual C2-domains of syt1 execute discrete functions during exocytosis, we created deletion mutants that lacked either the C2B domain (termed syt1-C2A) or the C2A domain (termed syt1-C2B) (Supp. Fig. 1A). These deletion constructs enabled examination of the remaining C2-domain in the context of the otherwise full-length protein. For functional analysis, these constructs were expressed in cortical neurons cultured from P0 – P1 syt1 KO mice.

**Fig. 1:**
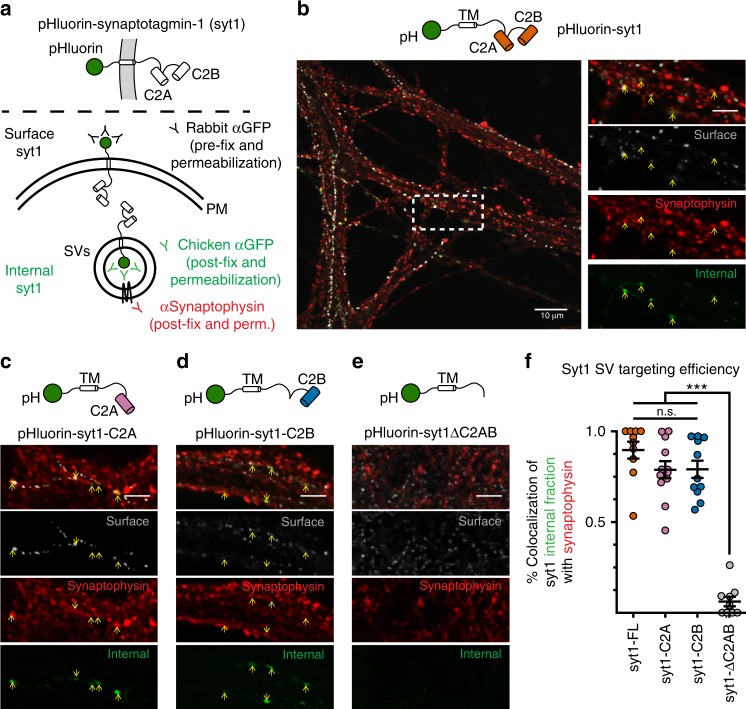
Either C2-domain of syt1 is sufficient for trafficking to synaptic vesicles. **a** Upper: constructs were tagged at the N-terminus with pHluorin and sparsely expressed in syt1 KO neurons. Lower: the tag enabled differential labeling of surface pHluorin from internalized pHluorin, using separate antibodies and a modified immunocytochemistry protocol. **b** Full-length syt1 (syt1-FL) was present both on the surface of the plasma membrane (gray) and in internal compartments (green). These internal compartments colocalized with a synaptic marker (synaptophysin, red), confirming that the fusion protein properly trafficked to synaptic vesicles. **c**, **d**. Syt1-C2A (**c**) and syt1-C2B (**d**) were also internalized (green) on synaptic vesicles, again marked by synaptophysin (red). **e** While a syt1 construct lacking both C2 domains trafficked to the surface (gray), this deletion mutant was seldom internalized (green). **f** Quantification of the colocalization of the internal fraction of each construct with synaptophysin. For panels **b** through **c**, scale bars in the zoomed images represent 5 µm. For all Figures, *p < 0.05, **p < 0.01; ***p < 0.001; n.s. indicates *p* > 0.05. Please see Supplementary Table 1 for the relevant data and statistics in this and all following data. Error bars represent s.e.m

We first examined whether the deletion constructs were properly targeted to SVs by appending an N-terminal pHluorin tag; this tag enabled a staining approach to distinguish the plasma membrane fraction from the internal fraction (Fig. 1a). Using this approach, we confirmed that full-length syt1 (syt1-FL) had both surface and internal fractions^32^, and that the internal fraction was highly colocalized with a SV marker (Fig. 1b, f, please see Supp. Table 1 for all data and statistics). Both syt1-C2A (Fig. 1c) and syt1-C2B (Fig. 1d) also had internal fractions that were highly colocalized with synaptophysin (Fig. 1f), suggesting that both constructs were properly targeted to SVs. Interestingly, a deletion mutant lacking both C2-domains (pH-syt1ΔC2A-B) had essentially no internal fraction and appeared to be stranded in the plasma membrane (Fig. 1e); the small amount of internal signal for this construct did not colocalize with the SV marker (Fig. 1f).

Next, we examined how efficiently these domain-deletion constructs targeted to boutons. Each construct was virally expressed (minus the pHluorin tag) in syt1 KO neurons at levels similar to the endogenous protein (Supp. Fig. 1B, C), and colocalization experiments examined the overlap of each construct with synaptophysin. Syt1-C2B accumulated in synaptic boutons equally well as syt1-FL (Supp. Fig. 1D). However, syt1-C2A had a significantly lower overlap with synaptophysin (Supp. Fig. 1D), indicating that sorting was impaired. Although C2B is not essential for targeting syt1 to at least some boutons, this domain ensures a broad distribution of syt1 to virtually all synapses.

### The C2B domain of syt1 is a clamp that inhibits SV fusion

**Fig. 2:**
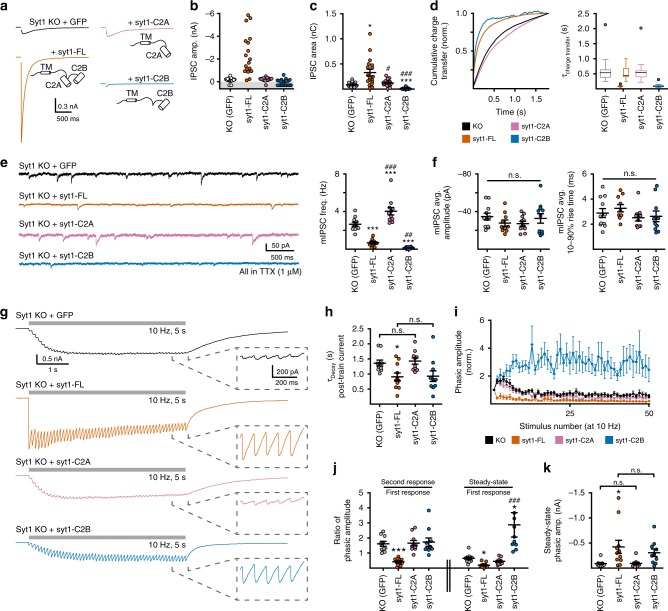
Individual C2-domains of syt1 differentially influence synaptic transmission. **a** Averaged evoked GABA_A_-receptor mediated IPSCs from syt1 KO neurons expressing a control virus (GFP, black), syt1-FL (orange), syt1-C2A (pink), or syt1-C2B (blue). **b**, **c** Quantification of the amplitude (**b**) and total charge (**c**) of the single-stimulation evoked IPSCs. In the amplitude graph (**b**), points in the gray shaded region of the graph indicate trials in which a response could not be evoked. **d** Graph (left) and quantification (right) of the cumulative charge transfer. Although neurons expressing syt1-FL required a double exponential, all other conditions were well fitted by a single exponential. The box plot is in the Tukey style: the center line is the median, the bounds are the first and third quartiles, and the whiskers are 1.5x the interquartile range. **e** Example traces (left) and quantification (right) of the frequency of spontaneous GABA_A_-receptor mediated mIPSCs in syt1 KO neurons expressing the indicated constructs. **f** Neither the amplitude (left) nor rise kinetics (right) of quantal responses were altered by the expression of the various syt1 constructs. **g** Average traces in response to train stimulation (10 Hz, 5 s) in syt1 KO neurons expressing the indicated constructs. Inset. Expanded view of the responses to the last 5 stimulations in the train. **h** Quantification of the post-train, delayed asynchronous component of release as fitted by a single exponential. **i** Quantification of the phasic amplitude (normalized to the first response) for each construct. **j** Quantification of the paired-pulse ratio (2nd response/1st response, left) and the steady-state ratio (average of the final 5 responses/1st response, right) for each construct. **k** Quantification of the steady-state amplitude for each condition. Note that while syt1-C2B expressing neurons initially have little release, they facilitate over the course of the train to near syt1-FL-mediated levels of release. Except where otherwise noted, stars (*) indicate statistical comparisons to neurons expressing the control virus; hash symbols (#) indicate statistical comparisons to neurons expressing syt1-FL. For all figures, #*p* < 0.05, ##*p* < 0.01; and ###*p* < 0.001. Error bars represent s.e.m

We next conducted electrophysiological recordings to determine whether and how these constructs influenced synaptic transmission. As expected, viral expression of syt1-FL rescued evoked synchronous release in KO neurons (Fig. 2a). This was evidenced by the increase in the amplitude (Fig. 2b) and total charge (Fig. 2c) of single-stimulation evoked GABA_A_-receptor mediated inhibitory postsynaptic currents (IPSCs). Furthermore, a sharp left-shift in the cumulative charge transfer (Fig. 2d) resulted from the appearance of a fast charge component that was missing in KO responses (Fig. 2d). Neither syt1-C2A nor syt1-C2B fully rescued synchronous release. In neurons expressing syt1-C2A, evoked IPSCs were no different than those recorded from KO neurons (Fig. 2a–d), so it is unlikely that the syt1-C2A construct regulated evoked fusion. The most striking finding was that syt1-C2B acted as a potent fusion clamp that strongly suppressed evoked neurotransmitter release (Fig. 2a–c). Moreover, the residual evoked release that occurred in the presence of syt1-C2B had the same kinetics as the fast component of release from neurons expressing syt1-FL (Fig. 2d). This clamping phenotype was not a by-product of the shortened spacing between C2B and the transmembrane domain, since inserting a flexible linker in place of C2A did not alter the syt1-C2B phenotype (Supp. Fig. 2).

Separately, we measured the frequency of mIPSCs in 1 μM tetrodotoxin (TTX). As expected, expression of syt1-FL in KO neurons reduced the frequency of mIPSCs (Fig. 2e). Here, expression of syt1-C2A significantly increased mIPSC frequency over the already elevated rate of KO neurons (Fig. 2e). Consistent with the clamping role observed in evoked IPSCs, expression of syt1-C2B sharply reduced the frequency of mIPSCs such that they were hardly observed (Fig. 2e). Neither syt1-C2A nor syt1-C2B altered the amplitude or shape of mIPSCs (Fig. 2f).

We also examined how KO neurons expressing these constructs responded to train stimulation (10 Hz, 5 s). KO neurons expressing syt1-FL depressed heavily over the entirety of the train (as indicated by the phasic amplitude, Fig. 2g, i, j) while KO neurons expressing the control virus initially facilitated (Fig. 2g, i, j). Moreover, expression of syt1-FL clamped the delayed asynchronous currents evident at the tail of the train (Fig. 2h). Syt1-C2A had no significant effect on the responses to train stimulation compared to the KO controls in terms of the phasic responses (Fig. 2i through K) or the delayed asynchronous currents (Fig. 2h). Interestingly, neurons expressing syt1-C2B continued to facilitate throughout the course of the train (Fig. 2i through K) such that, by the end of the train, the responses were similar in size and kinetics to those recorded in the syt1-FL condition (Fig. 2g, k). The increased paired-pulse ratio (PPR) in syt1-C2B expressing neurons suggests a sharp reduction in the probability of vesicle release (*P*_R_) as compared to syt1-FL expressing neurons (Fig. 2j). In addition, syt1-C2B clamped delayed asynchronous release to the same levels as syt1-FL (Fig. 2h).

**Fig. 3:**
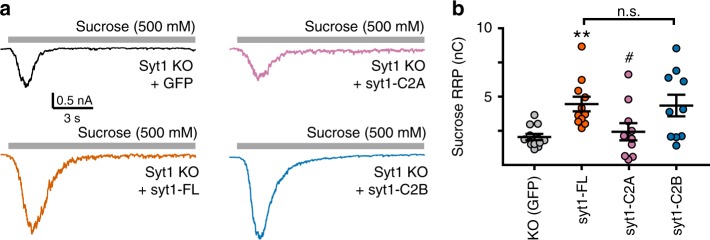
The syt1-C2B, but not syt1-C2A, rescues the RRP in syt1 KO neurons. Example traces (***a***) and quantification (***b***) of the readily releasable pool (RRP) elicited by the acute application of hypertonic sucrose (500 mM, 15 sec). The RRP was measured as the area under the curve prior to the onset of steady-state release (indicative of replenishment). Error bars represent s.e.m

Finally, we examined how each C2-domain contributed to the formation of the readily releasable pool of SVs (RRP). This pool is diminished in syt1 KO neurons^12,31^, and is rescued by expression of syt1-FL (Fig. 3a, b). Syt1-C2A did not rescue the RRP (Fig. 3a, b); in sharp contrast, syt1-C2B rescued the RRP to a similar degree as syt1-FL (Fig. 3a, b). Hence, C2B is necessary and sufficient to form the RRP, and the apparent clamping activity of this domain is not secondary to a loss of releasable vesicles. To further quantify this effect, we estimated the release probabilities (*P*_R_) by dividing the average total charge of an evoked IPSC by the average total charge of the RRP, yielding the following probabilities for each condition: 4.1 ± 0.7% in KO neurons, 7.3 ± 1.7% in syt1-FL expressing neurons, 5.0 ± 1.5% in syt1-C2A expressing neurons, and – remarkably – only 0.1 ± 0.1% in syt1-C2B expressing neurons (error values were estimated by propagating the s.e.m.). Qualitatively, these calculated *P*_R_ values are in general agreement with the PPR measurements (Fig. 2j).

### C2A and C2B must be in tandem to drive synchronous fusion

**Fig. 4:**
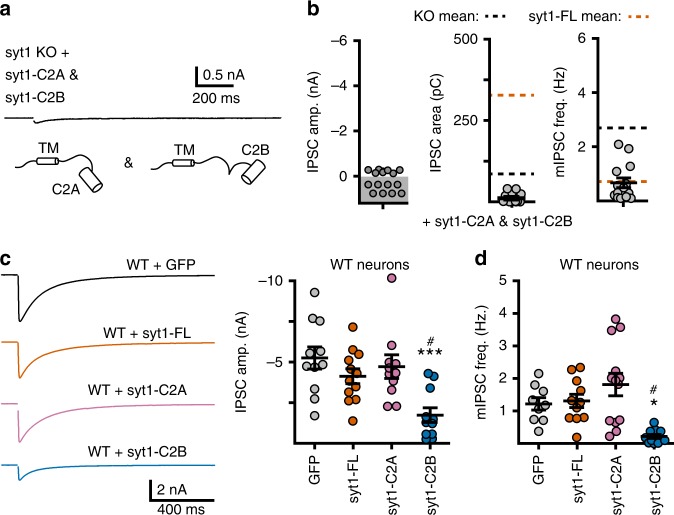
C2 domains must be configured in tandem for efficient evoked fusion. **a** Average trace illustrating evoked IPSCs from syt1 KO neurons co-expressing syt1-C2A and syt1-C2B. **b** Quantification of IPSC amplitude, IPSC charge, and mIPSC frequency illustrating that co-expression of these constructs does not mimic the overall function of the full-length protein. Note, data from panels **a** and **b** were collected in parallel with the data presented in Fig. 2; dashed lines on the IPSC area and the mIPSC frequency graph represent the mean values of these measurements for KO (black) and syt1-FL (orange) from the data presented in Fig. 2. **c** Left: average traces of evoked IPSCs recorded from WT neurons overexpressing a control virus (GFP, black, *n* = 11), syt1-FL (orange, *n* = 12), syt1-C2A (pink, *n* = 11), or syt1-C2B (blue, *n* = 11). Right: quantification of IPSC amplitudes. **d** Quantification of the frequency of mIPSCs from WT neurons overexpressing GFP (*n* = 9), syt1-FL (*n* = 11), syt1-C2A (*n* = 13), or syt1-C2B (*n* = 11). Error bars represent s.e.m

We next examined the functional significance of having tandem C2-domains, connected in the same parent protein, by co-expressing syt1-C2A and syt1-C2B in syt1 KO neurons. If these domains must be linked in order to function properly, co-expression should fail to mimic the activity of the full-length protein. Evoked release in these dual-expressing neurons was similar to evoked release in neurons expressing syt1-C2B alone; again, potent clamping activity was observed (Fig. 4a). The frequency of spontaneous release, in contrast, was elevated compared to KO neurons expressing syt1-C2B, but was depressed compared to KO neurons expressing syt1-C2A (Fig. 4b). We postulate that this intermediate phenotype is due to heterogeneous regulation of synaptic vesicles, with some vesicles being predominantly regulated by syt1-C2A while others are predominately regulated by syt1-C2B.

We also expressed the deletion constructs in otherwise WT neurons (Fig. 4c). For these experiments, more virus was applied to each culture, compared to experiments in syt1 KO neurons. Constructs were overexpressed at levels that were 5- to 10-fold higher than endogenous syt1; this did not alter the amount of native protein (Supp. Fig. 3A). We found that syt1-C2B reduced the amplitude of evoked release (Fig. 4c) and the frequency of spontaneous release events (Fig. 4d), similar to our findings using syt1 KO neurons. This suggests that syt1-C2B acts in a dominant negative manner and that the WT protein is unable to rescue or overcome the function of this deletion mutant. Furthermore, this finding provides additional evidence that the C2-domains must be linked in tandem for full function during spontaneous and evoked release. As in KO neurons, expression of syt1-C2A in WT neurons had no effect on evoked release (Fig. 4c).

Importantly, overexpression of syt1-FL in WT neurons did not alter the single-stimulation evoked response (Fig. 4c) or the frequency of miniature events (Fig. 4d). Separate localization experiments in syt1 KO neurons, utilizing the pH-syt1 construct (Fig. 1), show a drastic increase in plasma membrane localized fraction of syt1 when overexpressed at a similar level as in the functional experiments presented in Fig. 4c (Supp. Fig. 3B, C). The lack of any phenotype from syt1 overexpression, despite the greatly increased surface fraction, argues against a role for plasma membrane localized syt1 in clamping SV fusion.

### Syt1-C2A-driven minis do not require Ca^2+^-binding activity

**Fig. 5:**
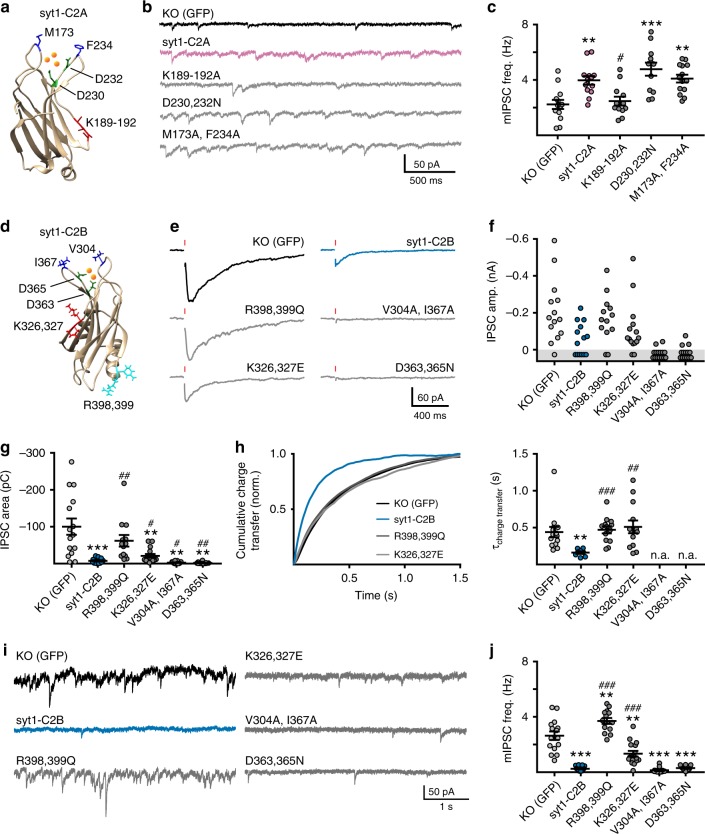
Mutagenesis of each C2-domain and impact on transmission. **a** Point mutations were introduced in the syt1-C2A domain-deletion construct. Crystal structure of C2A^34^ illustrating the locations of the mutants employed in this study. Ca^2+^ ions (orange) were drawn to illustrate the approximate Ca^2+^ binding sites. **b**, **c** Example traces (**b**) and quantification (**c**) of the frequency of mIPSCs in syt1 KO neurons expressing the indicated mutant construct of syt1-C2A. **d** Point mutations were also introduced in the syt1-C2B domain-deletion construct. NMR structure of C2B^73^; again, the position of each substitution is shown and Ca^2+^ ions (orange) were drawn to illustrate the approximate Ca^2+^ binding sites. **e**–**h** Average evoked IPSCs (**e**) and quantified amplitudes (**f**), total charges (**g**), and kinetics (**h**) for syt1 KO neurons expressing the indicated mutant construct of syt1-C2B. **i**, **j** Example traces (**i**) and quantification (**j**) of the rate of mIPSCs observed in syt1 KO neurons expressing the indicated constructs. Here, (*) indicates statistical comparisons to neurons expressing the control virus and (#) indicates statistical comparisons to neurons expressing WT syt1-C2A (***a***–***c****)* or WT syt1-C2B (**d**–**j**). Error bars represent s.e.m

To determine the mechanism by which syt1-C2A increased the frequency of minis (Fig. 2e), we introduced point mutations to selectively disrupt the following key features in this domain: the Ca^2+^-coordinating ligands (D230,232N)^33,34^, membrane-penetrating residues located at the tips of the Ca^2+^-binding loops (M173, F234A)^14^, and the poly-lysine patch (K189-192A)^35,36^ (Fig. 5a). None of these mutations alter expression or localization compared to syt1-C2A (Supp. Fig. 4). By expressing these mutants in KO neurons, we found that the syt1-C2A-mediated increase in mIPSC frequency required neither Ca^2+^-binding ligands nor membrane-penetrating residues (Fig. 5b, c). Rather, the poly-lysine patch was required for this effect (Fig. 5b, c). Thus, syt1-C2A did not increase the frequency of minis by canonical Ca^2+^-binding; rather, transient docking of SVs, mediated by the poly-lysine patch (please see Discussion) may explain the C2A phenotype.

### Distinct elements of syt1-C2B clamp and synchronize release

Analogous to the syt1-C2A experiments above, we also disrupted the Ca^2+^-coordinating residues (D363,365N)^37,38^, the membrane-penetration residues in the Ca^2+^-binding loops (V304A, I367A)^14^, and the poly-lysine patch (K326,327E)^14^ in syt1-C2B via mutations (Fig. 5d). In addition, arginine residues 398 and 399, thought to be important for binding t-SNARE heterodimers, were substituted to glutamine (R398,399Q)(Fig. 5d)^39,40^. These mutations did not alter expression or localization compared to syt1-C2B (Supp. Fig. 4).

In syt1 KO neurons, the potent clamping of evoked release by syt1-C2B (Fig. 5e) was disrupted in both the R398,399Q and K326,327E mutants. This was apparent from the larger percentage of responding neurons (Fig. 5f) and the increase in the average total charge (Fig. 5g). Notably, the R398,399Q mutant more severely disrupted clamping as evidenced by the larger increase in total evoked charge (Fig. 5g). Both mutants also disrupted the ability of syt1-C2B to synchronize evoked release (Fig. 5h). In contrast, KO neurons expressing either the Ca^2+^ ligand (D363,365N) or the membrane-penetration (V304A,I367A) mutant forms of syt1-C2B exhibited virtually no evoked SV fusion – synchronous or asynchronous (Fig. 5e through G). This finding indicates that the relatively low level of fully synchronous release depends on the canonical Ca^2+^-binding and membrane insertion activity of C2B.

The R398,399Q and K326,327E mutations also disrupted the ability of syt1-C2B to clamp spontaneous release, as evidenced by an increase in mini frequency (Fig. 5i, j). Interestingly, the R398,399Q mutant increased the frequency of spontaneous release above and beyond KO levels, similar to the phenotype of syt1-C2A (Fig. 5j). In contrast, neither the D363,365N nor the V304A,I367A mutations significantly altered the effect of syt1-C2B on mIPSC frequency (Fig. 5i, j), consistent with another Ca^2+^-sensor driving the majority of these miniature events^41^.

### Syt1-C2B clamping likely depends on interactions with SNAREs

**Fig. 6:**
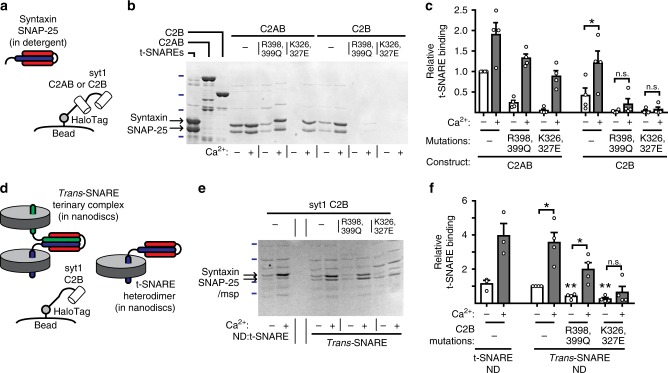
SNARE-binding activity correlates with the potent fusion clamp activity of C2B. **a** Soluble syt1C2AB or C2B was tagged with HALO, purified from bacteria, and conjugated to HALO resin beads. These beads were then used to pull-down full-length t-SNARE heterodimers. **b**, **c** Example gel (**b**), stained by Coomassie blue, with quantification (**c**) of the ability of each mutant construct to interact with t-SNARE heterodimers. Ladder marks (blue) indicated 25, 37, 50, and 75 kD standards. **d** HALO-tagged C2B, conjugated to beads, was used to pull-down *trans*-SNARE complexes or NDs bearing t-SNARE heterodimers. **e**, **f** Example gel (**e**), stained by Coomassie blue, with quantification (**f**) of the ability of each mutant to interact with the *trans*-SNARE complex. Ladder marks (blue) indicated 20, 25, 37, and 50 kD standards. Error bars represent s.e.m

Previous studies have reported that substitution of R398,399 and K326,327 disrupt Ca^2+^-independent binding of the isolated C2B domain to t-SNARE heterodimers^40^. Here, we also examined how these mutations, in a tandem C2 domain construct (C2AB) and in isolated C2B, affect SNARE interactions using a HaloTag-based pull-down assay. Purified HaloTag fusion constructs were covalently linked to HaloLink Resin beads (Supp. Fig. 5A, B) and used to pull-down t-SNARE heterodimers (SNAP-25B/syntaxin1a, Fig. 6a). As expected, robust Ca^2+^-independent binding was observed for WT C2AB, and this interaction was further enhanced by Ca^2+^ (Fig. 6b, c). Similar observations were made using isolated C2B, although the absolute degree of binding was less than for C2AB^14^. For both C2AB and C2B, R398,399Q and K326,327E mutations strongly impaired Ca^2+^-independent binding to t-SNAREs. Indeed, the mutant C2B domains exhibited no significant binding activity under these conditions (R398,399Q: *p* = 0.23, one sample *t*-test vs. 0; K326,327E: *p* = 0.30, one sample *t*-test vs. 0), consistent with a previous study^40^. Furthermore, while a strong Ca^2+^-dependent increase in binding was still observed for both mutants in the context of the tandem C2AB construct, the isolated C2B mutants again failed to bind SNAREs (Fig. 6b, c).

Next, we examined interactions between C2B and the *trans*-SNARE complex (Fig. 6d). In order to generate stable *trans*-SNARE complexes, t-SNAREs and v-SNAREs were reconstituted into separate populations of nanodiscs (NDs). These were then combined to form *trans*-SNARE complexes (i.e. partially zippered v- and t-SNAREs with their respective *trans*-membrane domains in opposing bilayers, Supp. Fig. 5C). Importantly, anionic phospholipids were omitted from the NDs, to avoid syt1-lipid interactions. NDs lacking SNARE proteins did not bind syt1 (Supp. Fig. 5C). WT C2B displayed robust Ca^2+^-independent binding to *trans*-SNARE NDs and this interaction was enhanced by Ca^2+^ (Fig. 6e). WT C2B pulled down t-SNARE heterodimers and *trans*-SNARE NDs equally well (Fig. 6e, f), so the presence of the v-SNARE does not strongly impact the t-SNARE binding activity of syt1. Furthermore, the R398,399Q and K326,327 mutations did not completely abolish Ca^2+^-independent *trans*-SNARE binding activity, although this component was significantly reduced in both mutants. Furthermore, a Ca^2+^-induced increase in *trans*-SNARE complex binding activity was observed for the R398,399Q construct, but not for the K326,327E mutant. The basis for this surprising observation is unclear and will require further study, but it is consistent with the idea that are multiple modes of binding between syt1 and SNAREs^42–45^.

### Complexin does not contribute to clamping by syt1-C2B

**Fig. 7:**
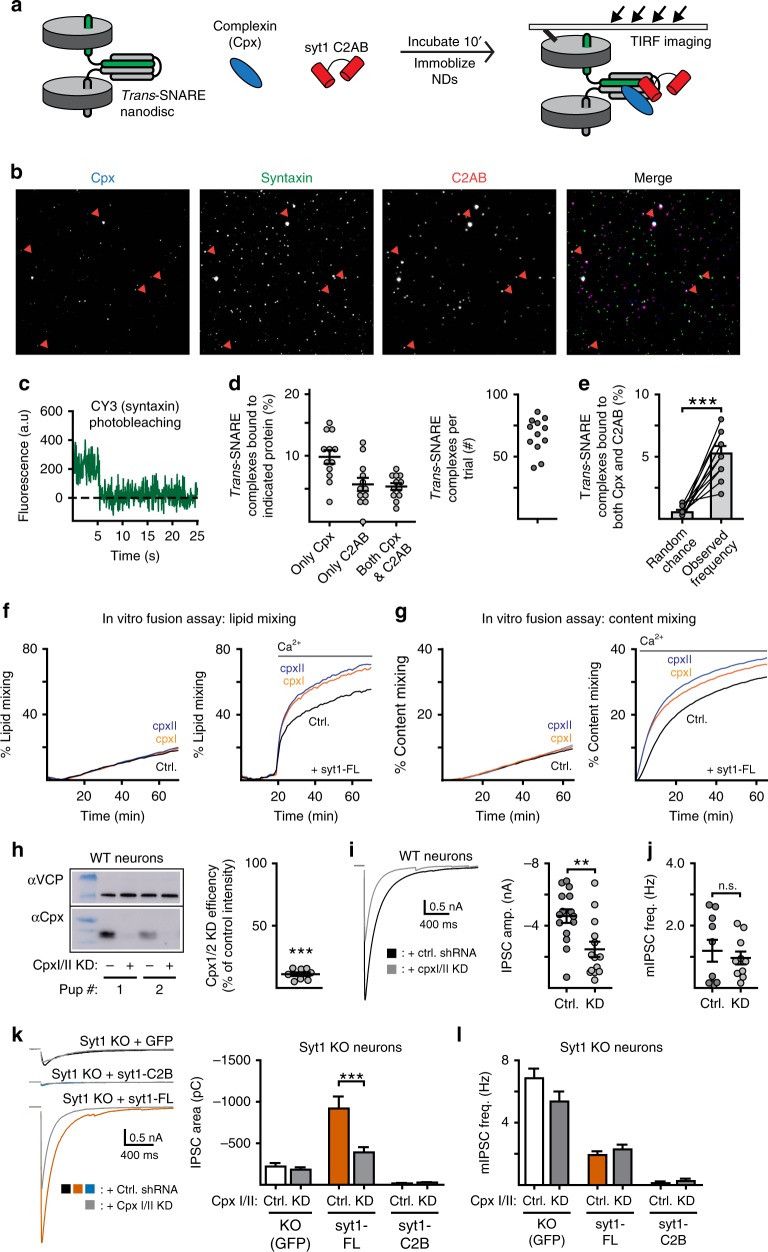
Complexin is not required for syt1-C2B-mediated clamping of fusion. **a** Illustration of the single-molecule imaging experiments. Binding was visualized via fluorescent dye labeling: syntaxin with CY3, the soluble C2AB domain of syt1 (C2AB) with CY5, and complexin (cpx) with Alexa Fluor 488. **b** Example image of the individual fluorophores corresponding to complexin (cpx; blue in the merge), syntaxin (green in the merge), and syt1 C2AB (C2AB; red in the merge). White puncta in the merge channel indicate the presence of all three molecules, dark blue puncta indicate syntaxin and cpx, and magenta puncta indicate syntaxin and C2AB. **c** Example trace when photobleaching the syntaxin channel. Nanodiscs (NDs) with only a single *trans*-SNARE complex were identified by a single step to zero fluorescence. **d** Left. Quantification of the frequency that *trans*-SNARE complexes were bound to either complexin alone, C2AB alone, or both. Right. Dot plot of the distribution of *trans*-SNARE complexes quantified per experiment. **e** The random chance of observing both C2AB and complexin bound to the same *trans*-SNARE complex, assuming independence between the binding events, was calculated by multiplying the individual binding frequencies that were observed in each experiment. Interestingly, the observed frequency of the *trans*-SNARE-C2AB-complexin complex was much higher than predicted by random chance. **f**, **g** In vitro fusion assays measuring lipid (**d**) and content (**e**) mixing. In both panels, the left graph shows the effect of complexin alone while the right graph demonstrates the effect of complexin when syt1 and Ca^2+^ were also present. **h** Immunoblot quantifying complexin protein levels for the KD experiments. **i** Average traces (left) and quantification (right) of the effect of cpx KD (gray) on single-stimulation evoked IPSCs recorded from WT neurons. **j** Quantification of the rate of miniature release in WT neurons with and without cpx KD. **k** Average traces (left) and quantification (right) of the effect of cpx KD (gray) in syt1 KO neurons expressing either a control virus (black/white), syt1-FL (orange), or syt1-C2B (blue). **l** Quantification of the effect of cpx KD on the rate of mIPSCs in the same expression conditions as **k**. Error bars represent s.e.m

A new C2B-SNARE binding mode was recently reported that shows C2B bound to a truncated *cis*-SNARE complex in conjunction with complexin; this tripartite binding interface was hypothesized to be the molecular basis of the fusion clamp^20^. As alluded to above, complexin has been proposed to clamp fusion by binding partially assembled SNARE complexes; then, Ca^2+^•syt1 displaces complexin to allow fusion to proceed^23^. However, it has also been reported that complexin and Ca^2+^•syt1 bind concurrently to SNAREs^24^. To examine whether complexin and syt1 bind concurrently or compete for binding to SNARE complexes, we used a single-molecule strategy. Importantly, we assayed for binding to *trans*-SNARE complexes, as all previous studies utilized *cis*-SNARE complexes which form only when all three SNAREs reside in the same membrane after fusion. To conduct these experiments, v- and t-SNAREs were again reconstituted into NDs to form *trans*-SNARE complexes. NDs bearing *trans*-SNARE complexes were incubated with the soluble C2AB domain of syt1 and complexin, in the presence of Ca^2+^, and then immobilized on a glass slide (Fig. 7a). Binding of C2AB and complexin to *trans*-SNAREs was visualized via three-color TIRF microscopy (Fig. 7a, b). To preclude the possibility of complexin and C2AB binding to separate SNARE complexes on the same ND, only single *trans*-SNARE complexes (determined by stochastic photobleaching of syntaxin; see Methods) were analyzed (Fig. 7c). While some of the NDs had multiple *trans*-SNARE complexes embedded in them, the majority had only one (Supp. Fig. 6). The key finding was that many of the individual *trans*-SNARE complexes associated with both syt1-C2AB and complexin (Fig. 7d), consistent with previously reported ensemble experiments that examined ternary *cis*-SNARE complexes^24,46^. Moreover, *trans*-SNARE complexes were significantly more likely to harbor both complexin and syt1-C2AB than predicted by chance; under these conditions, the frequencies of complexin alone and syt1-C2AB alone predict a mere 0.6 ± 0.1% chance of randomly finding both molecules bound to the same complex, in contrast to the observed 5.3 ± 0.5% occurrence (Fig. 7e). Thus, Ca^2+^•syt1 and complexin can concurrently bind to the same *trans*-SNARE complex and, interestingly, the concurrent binding of both molecules appears to be energetically favorable (Fig. 7e).

To address the functional interaction between *trans*-SNAREs, syt1, and complexin, we performed in vitro fusion assays. Previous studies have examined how the inclusion of complexin affects SNARE-driven membrane fusion in biochemical assays, in either the absence or presence of syt1. However, these studies report conflicting conclusions as to whether complexin stimulates^47^ or inhibits^46^ SNARE-driven fusion under various experimental conditions^24^. Here, we revisited this question by conducting fusion assays in the presence of a molecular crowder (Ficoll 70) to mimic the complex, crowded environment in synaptic boutons^48,49^. Addition of complexin I/II did not alter the rate of fusion of v- and t-SNARE proteoliposomes in the absence of syt1 and Ca^2+^ (Fig. 7f, g). Incorporation of full-length syt1 onto the v-SNARE bearing vesicles promoted fusion in a manner that was greatly enhanced by Ca^2+^ (Fig. 7f, g). Under this condition, inclusion of complexin now increased the rate and extent of fusion even beyond the levels achieved by syt1 alone (Fig. 7f, g).

To examine how this complex regulates fusion in synaptic boutons, complexin I/II were knocked-down (KD) using virally expressed shRNA. Complexin I/II KD was highly effective and reduced protein levels by ~90% (Fig. 7h). In WT neurons, complexin KD reduced the amplitude of evoked IPSCs by half (Fig. 7i). However, we observed no significant effect on the frequency of mIPSCs (Fig. 7j). Similar results were obtained from syt1 KO neurons rescued with syt1-FL (Fig. 7k, l and Supp. Fig. 6B, C). In marked contrast, KD of complexin had no observed effect in syt1 KO neurons; evoked IPSCs (Fig. 7k) and mIPSC frequency (Fig. 7l) were unchanged.

We then returned to the clamping activity of syt1-C2B expressed in syt1 KO neurons. Importantly, complexin was not required for the potent clamping phenotype of this domain. Though the total charge of evoked IPSCs was marginally increased by complexin KD (Fig. 7k), the degree of evoked fusion under both of these conditions was still only a small fraction of the release measured in syt1 KO neurons. Furthermore, the heavily depressed frequency of mIPSCs in neurons expressing syt1-C2B was unaffected by complexin KD (Fig. 7l). Thus, while there appear to be functional interactions between syt1-C2B and complexin during evoked release, these interactions are unnecessary for C2B-mediated clamping activity.

## Discussion

In this study, we determined whether the individual C2-domains of syt1 execute specific functions during SV exocytosis. Unexpectedly, we gained insights into a key question in synaptic neuroscience: since reconstituted SNARE proteins are constitutively active, what molecules clamp SNARE complexes to enable precisely timed control of SV fusion? Our results indicate that the C2B domain of syt1 corresponds to this long sought-after fusion clamp in mammalian nerve terminals.

We first found that either C2-domain was able to target syt1 to SVs^50^ (Fig. 1), using an approach that allowed the internal and surface fractions of syt1 deletion mutants to be independently visualized (Fig. 1a). Indeed, failing to do so confounded early attempts to localize these constructs^51^. Interestingly, a construct lacking both domains apparently traversed the secretory pathway to incorporate into the plasma membrane but was not internalized onto SVs (Fig. 1). We postulate that targeting to the plasma membrane represents the first step of the sorting pathway for syt1. Then, endocytic motifs located in either C2-domain^52^ would mediate incorporation into recycling SVs.

Neither syt1-C2A nor syt1-C2B was sufficient to fully rescue the syt1 KO phenotype^50^. However, each C2 domain did influence aspects of neurotransmission. Expression of syt1-C2A had no effect on evoked release in syt1 KO neurons, but increased the frequency of miniature events (Fig. 2). This phenotype did not require Ca^2+^ binding or membrane penetration (Fig. 5) and was not secondary to changes in the RRP (Fig. 3). Rather, it was abolished by substitutions to the poly-lysine motif (Fig. 5). We speculate that syt1-C2A may help to direct SVs to release sites via the poly-lysine patch. Because C2A alone cannot arrest fusion, these vesicles would fuse in an unregulated manner and cause the observed increase in mini frequency. Interestingly, the syt1-C2B R398,399Q mutant construct, which also was unable to arrest fusion and had an intact poly-lysine patch, mimicked syt1-C2A. These observations are consistent with the notion that syt1 assists in vesicle docking and priming^31,53–55^, though the molecular partners of these poly-lysine patches in this specific context have yet to be fully elucidated^14,36,56^.

Strikingly, syt1-C2B served as a powerful clamp that inhibited all forms of SV release (Fig. 2). This appears to be specific for mammalian neurons as this effect was not observed in *Drosophila* preparations^50^. The observation that syt1-C2B fully rescued the RRP in syt1 KO neurons (Fig. 3)^12,31^ emphasizes the potency of this apparent clamping function. Mutagenesis experiments correlated the clamping activity with Ca^2+^-independent C2B-SNARE interactions (Fig. 5). Interestingly, C2B associated equally well with *trans*-SNARE complexes and t-SNARE heterodimers (Fig. 6). So, *trans*-SNARE complexes and t-SNARE heterodimers may be equivalent targets for syt1, consistent with previous results examining truncated ternary *cis*-SNARE complexes^57,58^. Using a *trans*-SNARE interaction assay, we found that substitution of K326,327 or R398,399 in the C2B domain of syt1 impaired binding. However, unlike binding assays using t-SNARE heterodimers (Fig. 6a–c)^40^, some degree of *trans*-SNARE binding activity was still observed for both of these mutant constructs. The residual binding is consistent with the emerging view that C2B forms contacts with SNAREs via multiple surfaces, indicated by the distinct contact sites reported in different syt1-SNARE structures^42–45^. Multiple binding surfaces help unify the following two observations: (1) that the K326,327E and R398,399Q mutants were equally detrimental to overall Ca^2+^-independent *trans*-SNARE binding activity (Fig. 6), and (2) that the R398,399Q mutations were far more disruptive to the clamping function of C2B in neurons (Fig. 5). Indeed, the K326,327E mutant construct still displayed some degree of clamping activity as compared to the KO condition (Fig. 5). We interpret these results to indicate that the bottom-side of C2B (i.e. involving or near residues R389 and 399, located on the opposite end of the C2-domain relative to the Ca^2+^-binding loops)^45^ is the crucial surface by which C2B clamps the *trans*-SNARE complex. This idea is supported by the observation that bottom-side interactions between C2B and *cis*-SNARE complexes were abolished by substitutions at R398 and 399 and only mildly impaired by substitutions at K326 and 327^40^.

A recent crystal structure revealed a tripartite interface formed between the C2B domain of syt1, complexin, and a truncated *cis*-SNARE complex^20^. In this tripartite complex, complexin was hypothesized to be the fusion clamp that locks primed SVs in a fusion-competent state by inserting directly into SNARE complexes^20^. However, we found that the C2B-mediated fusion clamping did not require the presence of complexin I/II (Fig. 7). Moreover, KD of complexin did not increase the rate of spontaneous release under any condition examined (Fig. 7). These findings, and the resultant interpretation that complexin is not a fusion clamp in mammalian neurons, are in general agreement with numerous studies reporting a slight reduction^25,26,28,29^ or no change^22,27,28^ in the rate of minis in complexin KO synapses. Though another group reported a ~3 fold increase in mini frequency when complexin was knocked down in mouse neurons^17–20^, the fact that this observation was not reproduced in our KD experiments suggests that inherent differences in KD vs. KO methodologies are unlikely to be the root cause of this discrepancy^19^. Furthermore, in the context of the tripartite interface, C2B contacts SNAREs via a distinct interface that did not include the bottom-side residues of C2B^20^. Interestingly, this structure contained a separate binary complex that formed between the bottom-side of C2B and the SNARE complex, similar to the structure reported by Zhou et al. (2015)^45^. We propose that this binary complex^20,45^ represents the clamped SNARE complex, whereas the tripartite complex^20^ represents a fusogenic state. We find it intriguing that complexin clearly functions to limit fusion in invertebrate neurons^59,60^ and speculate that, over the course of evolution, the ability to clamp fusion may have migrated from complexin to the C2B domain of syt1. Indeed, the function of complexin is hypothesized to have evolved between invertebrates and mammals^15^ and, again, the C2B domain of syt1 does not appear to clamp fusion at the *Drosophila* neuromuscular junction^50^.

While syt1-C2B strongly clamped evoked fusion in syt1 KO mouse neurons, a small amount of evoked release was still observed. The kinetics of this residual release matched the fast component of evoked release seen in neurons expressing full-length syt1 (Fig. 2). Based on mutagenesis (Fig. 5), the low levels of C2B-mediated release required Ca^2+^-binding and membrane penetration activity, similar to the canonical activity of the full-length protein^38,51^. Thus, C2B appears to have multiple functions and is crucial for first arresting SVs and then facilitating synchronous fusion. Since the C2B domain was sufficient for arresting fusion (Fig. 2), forming or maintaining the RRP (Fig. 3), and generating some small amount of synchronous fusion (Fig. 2), it appears that the primary function of the C2A domain in SV exocytosis is to simply increase the reliability of syt1-mediated evoked transmission by increasing the probability of release. How C2A accomplishes this, however, remains unclear. That fact that the C2A domain must be configured in tandem with C2B (Fig. 4)^50^ suggests that direct interactions between these two domains may be required. Indeed, the C2 domains of syt1 are known to form direct contacts with each other^61–63^, and a dynamic change in the orientation between the C2 domains has been proposed to serve as a switch from an inhibited to an activated state^10,63^. Whether C2A must also bind Ca^2+^ is unclear due to uncertainty regarding the phenotypes of C2A Ca^2+^-ligand mutants. Neutralization of Ca^2+^-coordinating residues in C2A have been reported to have no effect, to be a gain of function, or to be a loss of function^64^. Tandem membrane penetration by both C2-domains may be required to sufficiently lower the energy barrier to generate robust fusion^64,65^.

In addition to C2A, complexin also promoted syt1-dependent synchronous exocytosis; KD of complexin I/II had no effect on evoked release in syt1 KO neurons (Fig. 7). Thus, while C2B does not require complexin to clamp fusion, complexin contributes to the efficiency of evoked release. One possibility is that complexin promotes syt1-mediated fusion via the identified tripartite interface, where complexin contacts both C2B and SNARE complexes^20^. Syt1 can promote folding of SNARE complexes^66^ and complexin favors at least partially assembled complexes^67^; so, their synergy during fusion likely involves changes in SNARE complex zippering.

## Methods

### Experimental model and cell culture

Cortical neuronal cultures were primarily prepared from postnatal day 0–1 syt1 KO mice (Jackson Laboratory; Stock #: 002478). Mice were maintained as heterozygous breeder pairs, and the gender of the cultured pups was not determined. As indicated for experiments related to Fig. 4c, d, cortical neurons were cultured from C56BL/6J mice or Sprague Dawley rat pups. All procedures were in accordance with relevant ethical regulations, under the guidelines of the National Institutes of Health, and approved by the Animal Care and Use Committee at the University of Wisconsin – Madison. In brief, cortices were dissected from mouse brain, digested for 20 min at 37 °C in 0.25% trypsin-EDTA (Corning), mechanically dissociated, and plated at a density of ~100,000 cells/cm^2^ onto 12 mm glass coverslips (Carolina Biological Supply) that were coated with poly-D-lysine. Cultures were grown in Neurobasal A medium (GIBCO) supplemented with B27 (2%, GIBCO) and GlutaMAX (2 mM, GIBCO) and maintained at 37 °C in a 5% CO_2_ humidified incubator.

For experiments involving lentivirus, DNA sequences encoding WT and mutant forms of syt1 were subcloned into a FUGW transfer plasmid modified with a synapsin promoter and an IRES-expressed soluble GFP marker. Lentiviral particles were generated as previously described^41^. In brief, HEK297T/17 cells were co-transfected with transfer and helper (pCD/NL-BH*ΔΔΔ and VSV-G encoding pLTR-G) plasmids. Lentivirus was collected from the media 48–72 h after transfection and concentrated by ultracentrifugation. This virus was used to infect neurons on day-in-vitro (DIV) 6. As monitored by the presence of the GFP marker, a >95% infection rate was achieved in all experiments.

For the localization experiments (Fig. 1), cortical neurons were sparsely transfected at DIV 5 with the indicated syt1 constructs using calcium phosphate. In brief, 4 µg of plasmid DNA in 250 mM CaCl_2_ was added dropwise, with brief vortexing after each drop, to an equal volume of 2x HBS (in mM: 275 NaCl, 10 KCl, 1 Na_2_HPO_4_, 15 D-glucose, and 40 HEPES pH 7.05). This mixture was incubated for 20 min at RT and then added to the media for one coverslip of cultured neurons. Neurons was then incubated for 45 min at 37 °C in 5% CO_2_. Afterwards, the media was exchanged for conditioned media pre-equilibrated to 10% CO_2_ and maintained at 37 °C in a 5% CO_2_ humidified incubator.

Complexin KD (Fig. 7) was achieved via commercially available lentiviruses that expressed shRNAs targeted against complexin I (Sigma, Cat. #: TRCN0000115106 // SHCLNV-NM_007756) or complexin 2 (Sigma, Cat. #: TRCN0000115104 // SHCLNV-NM_009946). A lentivirus expressing a non-targeted shRNA (Sigma, Cat. #: SHC002V) was used as a control in these experiments.

### Electrophysiology

Whole-cell voltage-clamp recordings were performed using a Multiclamp 700B amplifier (Molecular Devices) at DIV 14–19. Recordings were made at RT in a bath solutions containing (in mM): 128 NaCl, 5 KCl, 2 CaCl_2_, 1 MgCl_2_, 30 D-glucose, and 25 HEPES, pH 7.3 and 305 mOsm. Patch pipettes (3–5 ΜΩ) were pulled from borosilicate glass (Sutter Instruments) and the pipette internal solution contained (in mM): 130 KCl, 1 EGTA, 10 HEPES, 2 ATP, 0.3 GTP, and 5 sodium phosphocreatine, pH 7.35 and 275 mOsm. Data were acquired using a Digidata 1440A (Molecular Devices) and Clampex 10 software (Molecular Devices) at 10 kHz. Neurons were held at −70 mV. Series resistance was compensated and recordings were discarded if the access resistance rose above 15 ΜΩ at any point. GABA_A_ receptor mediated events were pharmacologically isolated by including D-AP5 (50 µM, Abcam) and CNQX (20 µM, Abcam) in the bath solution. GABA_A_ receptor mediated inhibitory postsynaptic currents (IPSCs) were examined to avoid disynaptic currents and because syt1 promotes inhibitory, but not excitatory, spontaneous events^41^. Recorded traces were analyzed using Clampfit 10 (Molecular Devices). In some experiments, neurotransmitter release was evoked by a single stimulus or train stimuli delivered via a concentric bipolar electrode (FHC, 125/50 µm extended tip). Stimulating electrodes were places ~100–200 µm away from the soma being recorded and stimulation currents (0.5–0.9 mA) were adjusted per recording to measure the maximum field-evoked current. In experiments measuring spontaneous release, tetrodotoxin (TTX, 1 µM) was included in the bath solution to inhibit action potentials. Sixty seconds of data were recorded for each cell and miniature events were identified in Clampfit using the template matching algorithm.

The readily releasable pool of vesicles (RRP) was measure by applying hypertonic sucrose (500 mM)^68^ via a fused silica needle (28 gauge, WPI) positioned ~500 µm away from the soma of the patched neuron. The sucrose solution was puffed with a Picospritzer III (Parker Hannifin). Sucrose was applied for 15 s, yielding a distinct fast and slow (steady-state) phase of release. The fast component was integrated to determine the RRP size.

### Immunocytochemistry

Immunocytochemistry was performed as previously described^41^, with one notable exception: immediately prior to permeabilization and fixation, dissociated cortical neurons were incubated for 5 min with a primary antibody directed against the pHluorin moeity (rabbit anti-GFP polyclonal; Abcam; 1:250 in culture medium). This primary antibody labeled only the extracellular-facing pHluorin tags as it could not enter neurons. After fixation and permeabilization, neurons were then incubated, a second time, with primary antibodies against synaptophysin (guinea pig, 1:500, Millipore) and pHluorin. This second, distinct pHluorin antibody (chicken anti-GFP monoclonal, 1:1000, abcam) only labeled copies of the tagged, expressed proteins that were not occluded by exposure to the first rabbit antibody, to selectively reveal the intracellular population. Primary antibodies were visualized using Alexa Fluor 488 conjugated goat anti-chicken, Alexa Fluor 568 conjugated goat anti-guinea pig, and Alexa Fluor 647 conjugated goat anti-rabbit conjugated secondary antibodies.

The experiments presented in Supp. Fig. 1D and Supp. Fig. 4 did not differentiate internal and surface fractions of syt1. Instead, total syt1 was labeled post fixation and permeabilization with an anti-synaptotagmin1 primary antibody (rabbit polyclonal, 1:500, Synaptic Systems) and visualized with Alexa Fluor 488 conjugated goat anti-rabbit. Synaptophysin was labeled as described above and visualize with Alexa Fluor 568.

Images were acquired on an Olympus FV1000 laser scanning confocal microscope, with a 60 × 1.4 NA oil immersion objective and PMT-based detection, using identical laser and gain settings for all samples. Images were analyzed and adjusted for brightness and contrast in ImageJ. For ICC analysis, an ‘n’ was considered to be a single field of view and n’s were collected from 2–3 separate dissections for each condition.

### Immunoblotting

Neuronal lysates were prepared by dissolving single neuronal coverslips in boiling lysis buffer (100 mM Tris-Cl, 200 mM DTT, 4% w/v SDS, 0.2% w/v bromophenol blue, 20% v/v glycerol, pH 6.8). Samples were run on 4–12% NuPage Bis-Tris gradient gels (Invitrogen) and transferred to nitrocellulose (GE) for blotting. For immunoblotting, syt1 was probed using 48.1 (mouse monoclonal, 1:500, 48.1), which recognizes the C2A domain of syt1. We also used a luminal domain antibody (rabbit polyclonal, 1:500, Synaptic Systems), which has a much stronger affinity for rat syt1 (i.e. the expression constructs used in this study) vs. the mouse protein. Additionally, blots were probed with an anti-complexin I/II (rabbit polyclonal, 1:1000, Cedarlane) and/or an anti-VCP antibody (mouse monoclonal, 1:1000, Abcam). Blots were visualized with HRP-conjugated secondary antibodies (goat anti-mouse IgG, 1:5000, Biorad). VCP was used as a loading control. Blots were imaged using an Amersham Imager 600 (GE) and brightness/contrast was adjusted for publication in ImageJ.

### Protein purification

For the HaloTag binding assays, constructs encoding syt1 C2AB (aa 96–421) and syt1 C2B (aa 273–421) were expressed with an N-terminal His_6_-HaloTag (pTrcHis A vector, ThermoFisher). These constructs were expressed in *E. coli*, purified via nickel-NTA chromatography, and eluted in His-tag elution buffer containing 500 mM imidazole, 400 mM KCl, 25 mM HEPES pH 7.4, and 5% glycerol. The SNAP-25B/syntaxin1a heterodimer was subcloned into the pRSF Duet vector (Novagen) with the His_6_-tag on the N terminus of SNAP-25, expressed in *E. coli*, purified via nickel-NTA chromatography, and eluted in His-tag elution buffer containing 1% octylglucoside and 2 mM DTT^41^.

For nanodisc (ND) based experiments, complexin, the cytosolic domain of syt1 (C2AB; residues 96–421), v-SNAREs, and t-SNAREs were expressed in and purified from *E. coli*^4,8,69^. NDs bearing single v-SNAREs (ND-v) or t-SNARE heterodimers (ND-t) were prepared using MSP1E3D1 as described previously^69,70^. 100% POPC lipids were used for ND-v, while 99% POPC and 1% biotin-PE were used for ND-t. NDs bearing *trans*-SNARE complexes were formed by incubating ND-t (2 µM) and ND-v (5 µM) overnight at 4 °C in reconstitution buffer; *trans*-complexes were then isolated on a sucrose gradient (10–20%). For in vitro fusion assays, full-length syt1, v-SNAREs, and t-SNARE heterodimers were reconstituted into liposomes as previously described^4,55,71^. For single-molecule imaging experiments, all cysteine residues were removed from syntaxin1a, syt1 C2AB, and complexin via mutagenesis. Then, cysteine residues were reintroduced at residue 203 of syntaxin1a, 234 of syt1 C2AB, and 131 of complexin; these cysteine residues were labeled with the fluorophores cy5, cy3, and Alex488, respectively. The labeling efficiency was determined to be ~0.8 mol of dye per mol of protein.

### HaloTag SNARE-binding assays

HaloTag SNARE-binding assays were conducted as previously described^41^. Briefly, purified His_6_-Halo tagged constructs were combined with HaloLink resin beads (100 µg protein with 100 µl bead volume), brought to 500 µl of total volume with binding buffer (150 mM KCl, 25 mM HEPES pH 7.4) and incubated for 30 min at RT with rotation. Complete binding of the HaloTag constructs to the bead was verified by SDS-PAGE (Supp. Fig. 5). Beads were washed 3x in binding buffer and then resuspended 1:1 in the same buffer. For detergent-based t-SNARE heterodimer binding assays, 40 µl of the 50% bead slurry was added to a 150 µl binding reaction containing 2.5 µM t-SNAREs (SNAP-25B/ syntaxin1a), 1 mM EGTA ± 1.5 mM Ca^2+^, 1 mM DTT, and 1% Triton X-100 in binding buffer. For binding assays using reconstituted t-SNARE NDs and *trans*-SNARE NDs, 20 µl of the 50% bead slurry was added to a 500-µl binding reaction containing 0.5 µM SNARE-bearing NDs and 1 mM EGTA ± 1.5 mM Ca^2+^ in binding buffer. In all assays, binding mixtures were incubated for 1 h at RT. Then, beads were washed 3x in binding buffer containing 1 mM EGTA ± 1.5 mM Ca^2+^, and bound SNAREs were eluted in 35 µl of 2x SDS sample buffer. For the detergent-based t-SNARE binding assays, 15 µl of eluate was subjected to SDS-PAGE; for the ND-based assays, all of the eluate was loaded onto gels. Gels were stained with Coomassie Blue, and the band intensities quantified in ImageJ with background subtraction and normalized to binding by the WT construct in EGTA.

### In vitro fusion assays

Lipid and content mixing assays, using v- and t-SNARE liposomes, were carried out as previously described^72^ except that the macromolecular crowding agent^49^, Ficoll 70 (100 mg/ml, GE Healthcare), was included in the reconstitution buffer (25 mM HEPES, 100 mM KCl, pH 7.4). Data were obtained from three independent trials.

### Single-molecule colocalization microscopy

Preparation of flow cells for single-molecule experiments was performed as described previously^69^. Purified *trans*-SNARE NDs (syntaxin1a labeled with cy3, 0.5 µM) were incubated with C2AB (labeled with cy5, 1 µM) and complexin I (labeled with Alexa Fluor 488, 1 µM) at RT for 10 min, and diluted to 10 pM before injection into flow cells. Unbound protein was washed out and samples were imaged in a buffer consisting of (in mM): 1 Trolox, 0.5 CaCl_2_, 100 KCl, and 25 HEPES pH 7.4, and an oxygen scavenging system (1% glucose, 1 mg/ml glucose oxidase, and 0.02 mg/ml catalase). Single-molecule imaging was performed using an Olympus IX83 inverted microscope equipped with a cellTIRF-4Line excitation system, a 60 × /1.49 Apo N objective (Olympus), and an Orca Flash4.0 CMOS camera (Hamamatsu Photonics, Skokie, IL). The following excitation filter sets: 488 nm, 590 nm, and 640 nm, were used to collect signals from Alexa Fluor 488, cy3, and cy5, respectively. Images were acquired using Metamorph and Olympus 7.8.6.0 (Molecular devices; Sunnyvale, CA), and adjusted for presentation in ImageJ.

### Materials and reagents

TTX, D-AP5, and CNQX were obtained from Abcam. Purified lipids used for reconstitution were obtained from Avanti Polar Lipids. Cell culture reagents were supplied by GIBCO and Atlanta Biological. Unless otherwise noted, other chemical reagents were obtained from Sigma.

### Quantification and statistical analysis

Values are reported as mean ± standard error of the mean (SEM). Graphically, bars represent the mean and error bars indicate the SEM. For electrophysiological experiments, each n represents a single recorded neuron and n’s were obtained from at least three separate animal preparations. All data were tested for normality; the appropriate statistical test was applied based on whether or not the data was normally distributed. All statistical tests were two-sided. Expected sample sizes were not estimated or predetermined. All statistical analysis was conducted using GraphPad Prism 7.01 (GraphPad Software).

### Reporting summary

Further information on research design is available in the Nature Research Reporting Summary linked to this article.

## Supplementary information

Supplementary Information

Peer Review

Reporting Summary

## Data Availability

Presented data can be found in the accompanying Supplementary Table 1; all other data available by request.

## Code availability

No custom software or analysis tools were used during this study. The molecular graphics for C2A and C2B were prepared with UCSF Chimera, developed by the Resource for Biocomputing, Visualization, and Informatics at the University of California, San Francisco, with support from NIH P41-GM103311.

## References

[1] Söllner T (1993). SNAP receptors implicated in vesicle targeting and fusion. Nature.

[2] Sutton RB, Fasshauer D, Jahn R, Brunger AT (1998). Crystal structure of a SNARE complex involved in synaptic exocytosis at 2.4 Å resolution. Nature.

[3] Weber T (1998). SNAREpins: minimal machinery for membrane fusion. Cell.

[4] Tucker WC, Weber T, Chapman ER (2004). Reconstitution of Ca2+-regulated membrane fusion by synaptotagmin and SNAREs. Science.

[5] Geppert M (1994). Synaptotagmin I: a major Ca2+ sensor for transmitter release at a central synapse. Cell.

[6] Littleton JT, Stern M, Schulze K, Perin M, Bellen HJ (1993). Mutational analysis of Drosophila synaptotagmin demonstrates its essential role in Ca2+-activated neurotransmitter release. Cell.

[7] Südhof TC, Rothman JE (2009). Membrane fusion: grappling with SNARE and SM proteins. Science.

[8] Chicka MC, Hui E, Liu H, Chapman ER (2008). Synaptotagmin arrests the SNARE complex before triggering fast, efficient membrane fusion in response to Ca2. Nat. Struct. Mol. Biol..

[9] Littleton JT, Stern M, Perin M, Bellen HJ (1994). Calcium dependence of neurotransmitter release and rate of spontaneous vesicle fusions are altered in Drosophila synaptotagmin mutants. Proc. Natl Acad. Sci. USA.

[10] Bai H (2016). Different states of synaptotagmin regulate evoked versus spontaneous release. Nat. Commun..

[11] Bello OD (2018). Synaptotagmin oligomerization is essential for calcium control of regulated exocytosis. Proc. Natl Acad. Sci. USA.

[12] Liu H, Dean C, Arthur CP, Dong M, Chapman ER (2009). Autapses and networks of hippocampal neurons exhibit distinct synaptic transmission phenotypes in the absence of synaptotagmin I. J. Neurosci..

[13] Wierda KDB, Sørensen JB (2014). Innervation by a GABAergic neuron depresses spontaneous release in glutamatergic neurons and unveils the clamping phenotype of synaptotagmin-1. J. Neurosci..

[14] Chapman ER (2008). How does synaptotagmin trigger neurotransmitter release?. Annu. Rev. Biochem..

[15] Trimbuch T, Rosenmund C (2016). Should I stop or should I go? The role of complexin in neurotransmitter release. Nat. Rev. Neurosci..

[16] Kaeser-Woo YJ, Yang X, Südhof TC (2012). C-terminal complexin sequence is selectively required for clamping and priming but not for Ca2+ triggering of synaptic exocytosis. J. Neurosci..

[17] Maximov A, Tang J, Yang X, Pang ZP, Südhof TC (2009). Complexin controls the force transfer from SNARE complexes to membranes in fusion. Science.

[18] Yang X, Kaeser-Woo YJ, Pang ZP, Xu W, Südhof TC (2010). Complexin clamps asynchronous release by blocking a secondary Ca2+ sensor via its accessory α helix. Neuron.

[19] Yang X, Cao P, Südhof TC (2013). Deconstructing complexin function in activating and clamping Ca2 + -triggered exocytosis by comparing knockout and knockdown phenotypes. Proc. Natl Acad. Sci. USA.

[20] Zhou Q (2017). The primed SNARE-complexin-synaptotagmin complex for neuronal exocytosis. Nature.

[21] Giraudo CG, Eng WS, Melia TJ, Rothman JE (2006). A clamping mechanism involved in SNARE-dependent exocytosis. Science.

[22] Trimbuch T (2014). Re-examining how complexin inhibits neurotransmitter release. eLife.

[23] Tang J (2006). A complexin/synaptotagmin 1 switch controls fast synaptic vesicle exocytosis. Cell.

[24] Chicka MC, Chapman ER (2009). Concurrent binding of complexin and synaptotagmin to liposome-embedded SNARE complexes. Biochemistry.

[25] Chang S (2015). Complexin stabilizes newly primed synaptic vesicles and prevents their premature fusion at the mouse calyx of held synapse. J. Neurosci..

[26] Lin M-Y (2013). Complexin facilitates exocytosis and synchronizes vesicle release in two secretory model systems. J. Physiol..

[27] Reim K (2001). Complexins regulate a late step in Ca2+-dependent neurotransmitter release. Cell.

[28] Xue M (2008). Complexins facilitate neurotransmitter release at excitatory and inhibitory synapses in mammalian central nervous system. Proc. Natl Acad. Sci. USA.

[29] Xue M (2010). Binding of the complexin N terminus to the SNARE complex potentiates synaptic vesicle fusogenicity. Nat. Struct. Mol. Biol..

[30] López-Murcia FJ, Reim K, Jahn O, Taschenberger H, Brose N (2019). Acute complexin knockout abates spontaneous and evoked transmitter release. Cell Rep..

[31] Chang S, Trimbuch T, Rosenmund C (2018). Synaptotagmin-1 drives synchronous Ca2+ triggered fusion by C2B domain-mediated synaptic vesicle-membrane attachment. Nat. Neurosci..

[32] Fernández-Alfonso T, Kwan R, Ryan TA (2006). Synaptic vesicles interchange their membrane proteins with a large surface reservoir during recycling. Neuron.

[33] Shao X, Davletov BA, Sutton RB, Südhof TC, Rizo J (1996). Bipartite Ca2+-binding motif in C2 domains of synaptotagmin and protein kinase C. Science.

[34] Sutton RB, Davletov BA, Berghuis AM, Sudhof TC, Sprang SR (1995). Structure of the first C2 domain of synaptotagmin I: a novel Ca2+/phospholipid-binding fold. Cell.

[35] Mace KE, Biela LM, Sares AG, Reist NE (2009). Synaptotagmin I stabilizes synaptic vesicles via its C2A polylysine motif. Genesis.

[36] Takahashi H, Shahin V, Henderson RM, Takeyasu K, Edwardson JM (2010). Interaction of synaptotagmin with lipid bilayers, analyzed by single-molecule force spectroscopy. Biophys. J..

[37] Earles CA, Bai J, Wang P, Chapman ER (2001). The tandem C2 domains of synaptotagmin contain redundant Ca2+ binding sites that cooperate to engage t-SNAREs and trigger exocytosis. J. Cell Biol..

[38] Nishiki T, Augustine GJ (2004). Dual roles of the C2B domain of synaptotagmin I in synchronizing Ca2+-dependent neurotransmitter release. J. Neurosci..

[39] Gaffaney JD, Dunning FM, Wang Z, Hui E, Chapman ER (2008). Synaptotagmin C2B domain regulates Ca2+-triggered fusion in vitro. J. Biol. Chem..

[40] Wang S, Li Y, Ma C (2016). Synaptotagmin-1 C2B domain interacts simultaneously with SNAREs and membranes to promote membrane fusion. eLife.

[41] Courtney NA, Briguglio JS, Bradberry MM, Greer C, Chapman ER (2018). Excitatory and inhibitory neurons utilize different Ca2+ sensors and sources to regulate spontaneous release. Neuron.

[42] Dai H, Shen N, Araç D, Rizo J (2007). A quaternary SNARE–synaptotagmin–Ca2+–phospholipid complex in neurotransmitter release. J. Mol. Biol..

[43] Choi UB (2010). Single-molecule FRET–derived model of the synaptotagmin 1–SNARE fusion complex. Nat. Struct. Mol. Biol..

[44] Brewer KD (2015). Dynamic binding mode of a Synaptotagmin-1–SNARE complex in solution. Nat. Struct. Mol. Biol..

[45] Zhou Q (2015). Architecture of the synaptotagmin–SNARE machinery for neuronal exocytosis. Nature.

[46] Schaub JR, Lu X, Doneske B, Shin Y-K, McNew JA (2006). Hemifusion arrest by complexin is relieved by Ca^2+^–synaptotagmin I. Nat. Struct. Mol. Biol..

[47] Malsam J (2009). The carboxy-terminal domain of complexin I stimulates liposome fusion. Proc. Natl Acad. Sci. USA.

[48] Homouz D, Perham M, Samiotakis A, Cheung MS, Wittung-Stafshede P (2008). Crowded, cell-like environment induces shape changes in aspherical protein. Proc. Natl Acad. Sci. USA.

[49] Yu H (2015). Reconstituting intracellular vesicle fusion reactions: the essential role of macromolecular crowding. J. Am. Chem. Soc..

[50] Lee J, Guan Z, Akbergenova Y, Littleton JT (2013). Genetic analysis of synaptotagmin C2 domain specificity in regulating spontaneous and evoked neurotransmitter release. J. Neurosci..

[51] Yao J, Kwon SE, Gaffaney JD, Dunning FM, Chapman ER (2011). Uncoupling the roles of synaptotagmin I as a dual Ca2+ sensor during endo- and exocytosis of synaptic vesicles. Nat. Neurosci..

[52] Lou X (2018). Sensing exocytosis and triggering endocytosis at synapses: synaptic vesicle exocytosis–endocytosis coupling. Front. Cell. Neurosci..

[53] Loewen CA, Lee S-M, Shin Y-K, Reist NE (2006). C2B polylysine motif of synaptotagmin facilitates a Ca2+-independent stage of synaptic vesicle priming in vivo. Mol. Biol. Cell.

[54] Reist NE (1998). Morphologically docked synaptic vesicles are reduced in synaptotagmin mutants of drosophila. J. Neurosci..

[55] Wang Z, Liu H, Gu Y, Chapman ER (2011). Reconstituted synaptotagmin I mediates vesicle docking, priming, and fusion. J. Cell Biol..

[56] de Wit H (2009). Synaptotagmin-1 docks secretory vesicles to syntaxin-1/SNAP-25 acceptor complexes. Cell.

[57] Lai Y, Lou X, Diao J, Shin Y-K (2015). Molecular origins of synaptotagmin 1 activities on vesicle docking and fusion pore opening. Sci. Rep..

[58] Bai J, Wang C-T, Richards D, Jackson MB, Chapman ER (2004). Fusion pore dynamics are regulated by synaptotagmin•t-SNARE interactions. Neuron.

[59] Cho RW, Song Y, Littleton JT (2010). Comparative analysis of Drosophila and mammalian complexins as fusion clamps and facilitators of neurotransmitter release. Mol. Cell. Neurosci..

[60] Xue M (2009). Tilting the balance between facilitatory and inhibitory functions of mammalian and drosophila complexins orchestrates synaptic vesicle exocytosis. Neuron.

[61] Evans CS (2016). Functional analysis of the interface between the tandem C2 domains of synaptotagmin-1. Mol. Biol. Cell.

[62] Fuson KL, Montes M, Robert JJ, Sutton RB (2007). Structure of human synaptotagmin 1 C2AB in the absence of Ca2+ reveals a novel domain association. Biochemistry.

[63] Liu H (2014). Linker mutations dissociate the function of synaptotagmin I during evoked and spontaneous release and reveal membrane penetration as a step during excitation-secretion coupling. Nat. Neurosci..

[64] Chapman ER (2018). A Ca2+ sensor for exocytosis. Trends Neurosci..

[65] Martens S, Kozlov MM, McMahon HT (2007). How synaptotagmin promotes membrane fusion. Science.

[66] Bhalla A, Chicka MC, Tucker WC, Chapman ER (2006). Ca2+–synaptotagmin directly regulates t-SNARE function during reconstituted membrane fusion. Nat. Struct. Mol. Biol..

[67] McMahon HT, Missler M, Li C, Südhof TC (1995). Complexins: cytosolic proteins that regulate SNAP receptor function. Cell.

[68] Rosenmund C, Stevens CF (1996). Definition of the readily releasable pool of vesicles at hippocampal synapses. Neuron.

[69] Lou X, Shin J, Yang Y, Kim J, Shin Y-K (2015). Synaptotagmin 1 is an antagonist for Munc18-1 in SNARE-zippering. J. Biol. Chem..

[70] Bao H (2016). Exocytotic fusion pores are composed of both lipids and proteins. Nat. Struct. Mol. Biol..

[71] Bao H (2018). Dynamics and number of *trans*-SNARE complexes determine nascent fusion pore properties. Nature.

[72] Hui E (2011). Mechanism and function of synaptotagmin-mediated membrane apposition. Nat. Struct. Mol. Biol..

[73] Fernandez I (2001). Three-dimensional structure of the synaptotagmin 1 C2B-domain: synaptotagmin 1 as a phospholipid binding machine. Neuron.

