# Supplementary material for: Synaptotagmin 1 clamps synaptic vesicle fusion in mammalian neurons independent of complexin
Source: Nat Commun. 2019 Sep 9;10:4076. doi: 10.1038/s41467-019-12015-w (PMC6733930; doi:10.1038/s41467-019-12015-w)
Supplement: Supplementary file 1 — Supplementary Information [file 41467_2019_12015_MOESM1_ESM.pdf]

Supplementary Figures

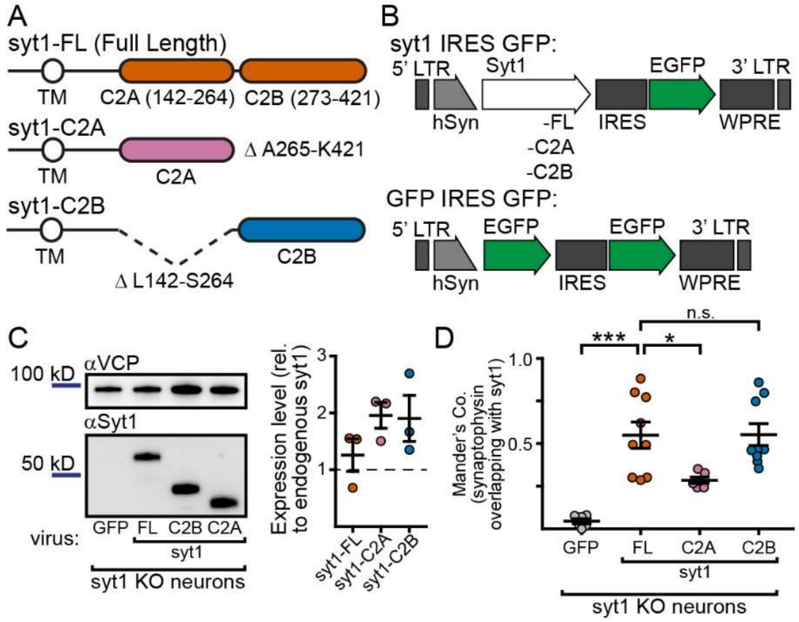

Supplementary Figure 1, related to Figure 1: Syt1 domain deletion construct maps, expression levels, and localization.

A. Illustration of each C2-domain deletion construct. TM indicates the transmembrane domain; the C2 domains are labeled C2A and C2B. Dotted lines indicate where constructs were fused together. *B*. Illustration of the lentiviral vector used to express the domain deletion constructs. *C*. *Left.* Immunoblot of syt1 KO neurons expressing the indicated construct using antibodies against the luminal domain of syt1; VCP served as a loading control. *Right.* Quantification of the amount of virally expressed constructs relative to the levels of endogenous syt1. *D*. Mander's analysis quantifying the overlap of synaptophysin with each indicated construct. For these experiments, constructs were virally expressed in syt1 KO neurons. Data were acquired in parallel with the data presented in Supp. Fig. 4C, though separate analysis was conducted. For representative images, please see Supp. Fig. 4C. Error bars represent s.e.m.

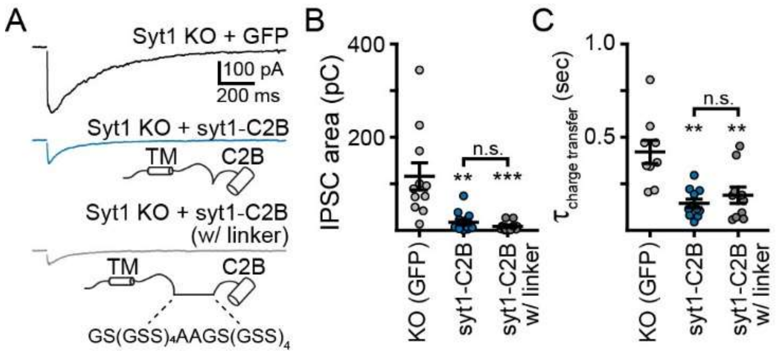

Supplementary Figure 2, related to Figure 2: The syt1-C2B phenotype is not due to shortening of the juxtamembrane linker.

A. Average traces of single stimulation evoked IPSCs from syt1 KO neurons expressing a control virus (top, n = 11), syt1-C2B (middle, n = 14), or syt1-C2B with an extended linker region (bottom, n = 12). To extend this linker (bottom, insert), a flexible segment comprised of glycine, serine, and alanine residues was inserted. *B and C*. Insertion of this flexible linker in syt1-C2B had no effect on the area (*B*) or kinetics (*C*) of single stimulation evoked IPSCs, as compared to syt1-C2B. Error bars represent s.e.m.

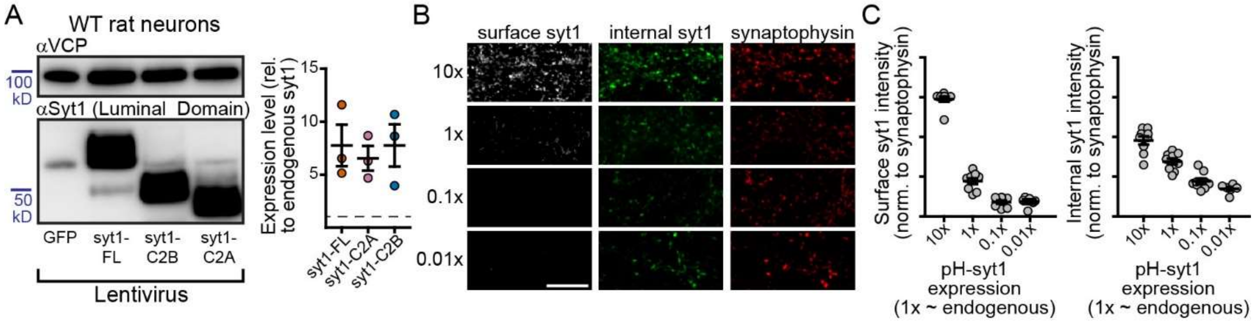

Supplementary Figure 3, related to Figure 4: The plasma membrane fraction of syt1 greatly increases upon over-expression.

A. Left. Representative immunoblot demonstrating that overexpression of the domain-deletion constructs does not alter endogenous levels of syt1 in WT neurons. Note, cultured rat neurons were utilized for this example blot so that each band of interest (i.e. the endogenous protein and all three viral constructs) could be visualized, and easily compared, using a single antibody approach. Similar results were obtained in WT mouse neurons by comparing blots using multiple syt1 antibodies. *Right.* Quantification of the amount of overexpressed syt1 constructs relative to the level of endogenous syt1. *B*. Representative images, from syt1 KO neurons, illustrating that over-expression of syt1-FL causes a sharp increase in the plasma-membrane localized fraction (surface, white), while the internal fraction of syt1 (green) is only slightly increased. Synaptophysin (red) was used as a synaptic marker. Scale bar represents 10 microns and applies to all images. *C.* Images were quantified by the overall intensity of the surface or internal fraction of syt1 and normalized to number of synapses in the field of view (approximated by the intensity of synaptophysin). For both *B* and *C*, the 1x condition corresponds to the amount of virus that results in syt1 expression equal to 1.15 ± 0.14 (n = 3) times the endogenous levels; the 10x, 0.1x, and 0.01x condition correspond to neurons infected with that many times more or less virus. Error bars represent s.e.m.

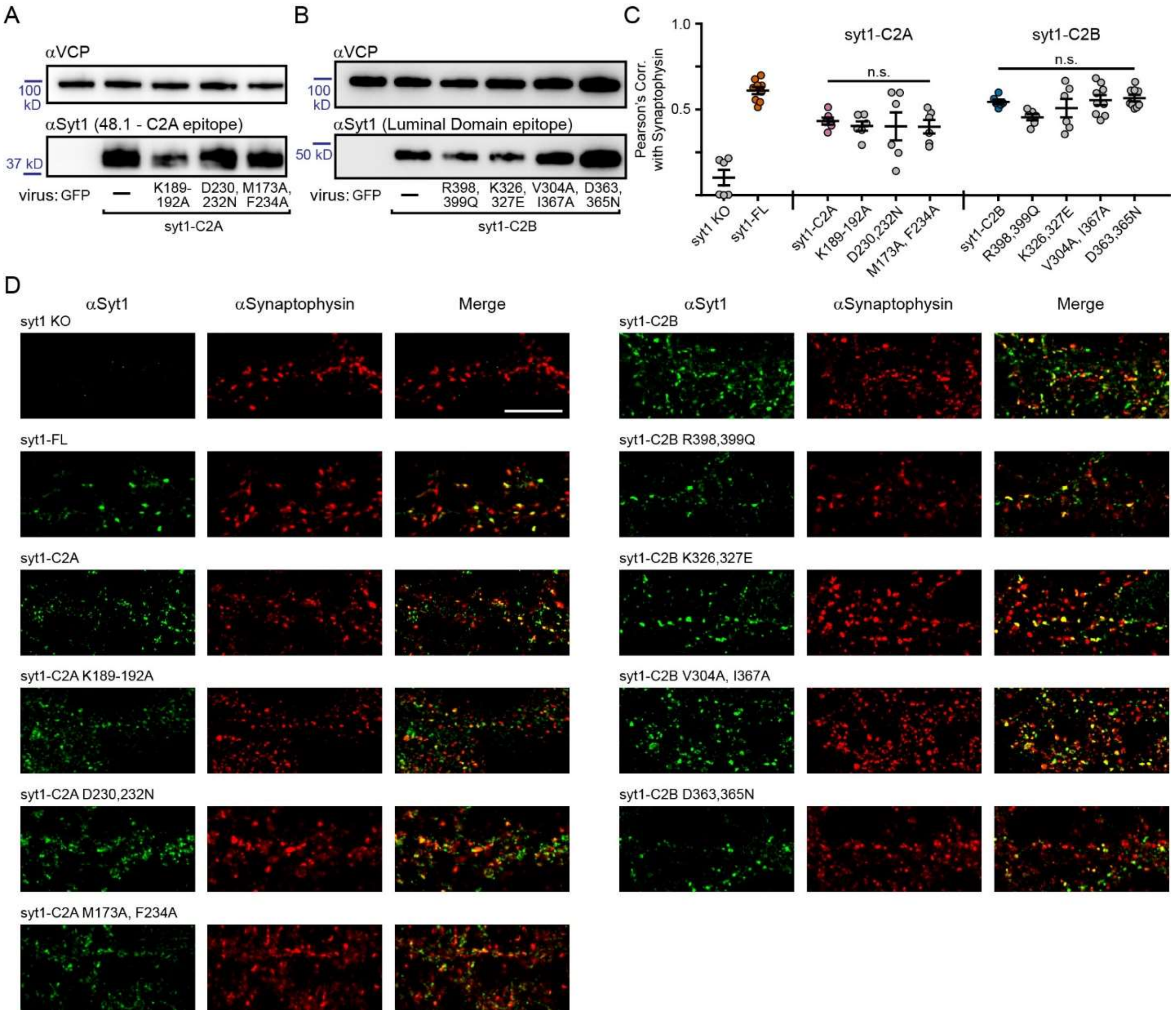

Supplementary Figure 4, related to Figure 5: Point mutations in syt1-C2A and syt1-C2B do not alter expression or localization.

A and B. Representative immunoblots demonstrating robust expression of all point-mutants. In both panels, the dash indicates the un-mutated, domain-deletion construct. *C*. Quantification of the Pearson's correlation between the point-mutants and a synaptic marker (synaptophysin). The localization of neither syt1-C2A nor syt1-C2B was altered by point mutations, as determined by an ANOVA. *D*. Representative images of the data quantified in *C*. Scale bar (found in syt1 KO, Merge) represents 10 μm and applies to all images. Error bars represent s.e.m.

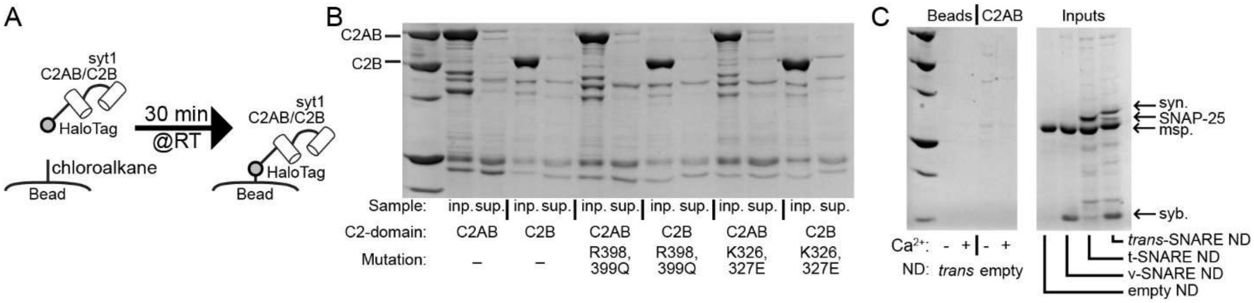

Supplementary Figure 5, related to Figure 6: Purified C2AB and C2B were efficiently absorbed onto to HALO resin beads.

A. C2-domain constructs (C2AB or C2B) where tagged at the N-terminus with HALO and purified from bacteria. Then, they were incubated for 30 minutes at RT with HALO resin beads (100 µg protein to 100 µl beads). *B.* After incubation, the supernatants (sup.; 2% of total) and the input protein (inp.; 2 µg; 2% of total input) were subjected to SDS-PAGE and proteins were visualized by staining with Coomassie blue. In all cases, the 30 minute incubation period was sufficient to completely deplete the supernatant of the C2 domain construct, indicating that all C2-domain protein was bound to the beads. *C*. Left. Example gel of the ND assay control experiments. Blank beads did not bind *trans*-SNARE NDs, and WT C2AB, on beads, did not bind NDs that lacked SNARE proteins. Right. Example gel of the inputs for the ND binding assays (25% of the total input).

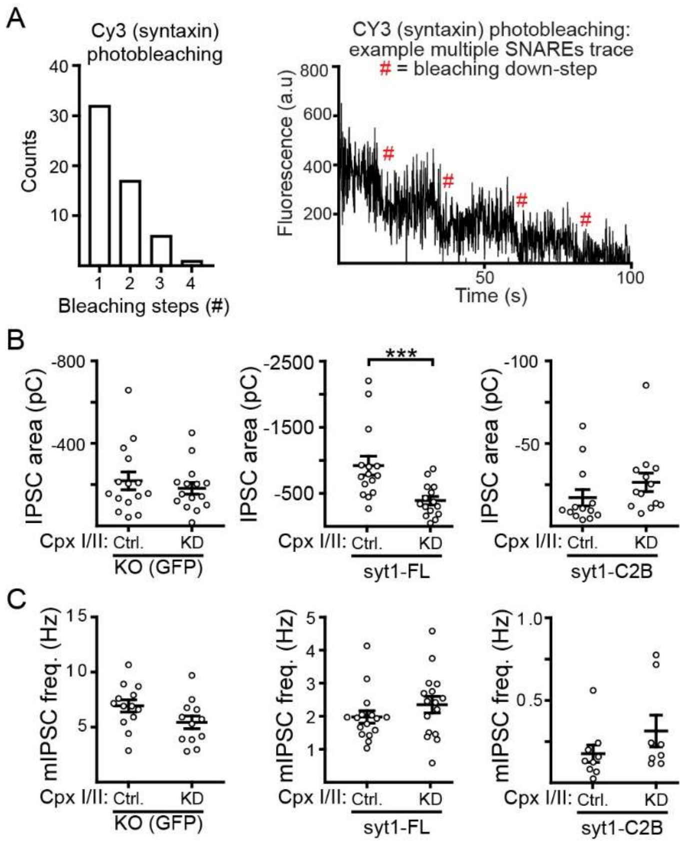

Supplementary Figure 6, related to Figure 7: Expanded data for single molecule photobleaching experiments and complexin KD electrophysiological experiments.

A. Left. Histogram depicting the number of down-steps observed per puncta when photobleaching Cy3 (labeling syntaxin). These data indicate that the majority of the nanodiscs (NDs) contained a single *trans*-SNARE complex. *Right.* Example trace demonstrating a nanodisc that had 4 *trans*-SNARE copies (i.e. 4 down-steps in the fluorescent trace). *B and C*. Data from Fig. 7I (*B*) and Fig. 7J (*C*) replotted on expanded scales. Error bars represent s.e.m.

Supplemental Table 1: Data Table

Figure	Parameter	Experimental System	Condition (LV expression or Purified Protein)	Mean	SEM	N	Statistical Test	P-value (Multiple Comparison)	P-Values (Indicated Comparisons)
1F	Manders Colocalization (internal fraction with synaptophysin) (%)	syt1 KO neurons	syt1-FL	89	5	10	ANOVA; Dunnett's Post-hoc	<0.001	n/a
			syt1-C2A	79	5	12			0.21 vs. syt1-FL
			syt1-C2B	80	5	11			0.23 vs. syt1-FL
			syt1-deltaC2AB	6	3	10			<0.001 vs. syt1-FL
2B	IPSC Amplitude (pA), Average of Successes Only	syt1 KO neurons	KO (GFP)	-203	31	16	Kruskal-Wallas; Dunn's Post-hoc	<0.001	n/a
			syt1-FL	-2392	431	18			< 0.001 vs. KO
			syt1-C2A	-238	26	15			0.40 vs. KO; 0.003 vs. syt1-FL
			syt1-C2B	-148	51	11			0.47 vs. KO; <0.001 vs. syt1-FL
2B	IPSC Failures (Count)	syt1 KO neurons	KO (GFP)	2	n/a	18	Student's T-Test		n/a
			syt1-FL	0	n/a	18			n/a
			syt1-C2A	1	n/a	16			n/a
			syt1-C2B	10	n/a	21			0.018 vs. KO
2C	IPSC Area (pC)	syt1 KO neurons	KO (GFP)	85	11	18	Kruskal-Wallas; Dunn's Post-hoc	<0.001	n/a
			syt1-FL	327	67	18			0.002 vs. KO
			syt1-C2A	122	18	16			0.09 vs. KO
			syt1-C2B	8	3	21			< 0.001 vs. KO; <0.001 vs. syt1-FL
2D	τ Charge Transfer (sec)	syt1 KO neurons	KO (GFP)	0.62	0.1	17	Kruskal-Wallas; Dunn's Post-hoc	<0.001	n/a
			syt1-FL (fast)	0.09	0.01	15			<0.001 vs. KO
			syt1-FL (slow)	0.53	0.06	15			0.99 vs. KO
			syt1-C2A	0.61	0.1	15			0.94 vs. KO
			syt1-C2B	0.11	0.2	10			< 0.001 vs. KO; 0.53 vs. syt1-FL [fast]
2E	mIPSC Frequency (Hz)	syt1 KO neurons	KO (GFP)	2.7	0.2	11	ANOVA; Sidak's Post-hoc	<0.001	n/a
			syt1-FL	0.7	0.1	10			< 0.001 vs. KO
			syt1-C2A	4.0	0.4	11			0.005 vs. KO; <0.001 vs. syt1-FL
			syt1-C2B	0.1	0.1	12			< 0.001 vs. KO; <0.001 vs. syt1-FL
2F	mIPSC Amplitude (pA)	syt1 KO neurons	KO (GFP)	-35	4	11	ANOVA	0.42	n/a
			syt1-FL	-28	3	10			n/a
			syt1-C2A	-27	2	11			n/a
			syt1-C2B	-33	5	11			n/a
2F	mIPSC 10-90% Rise Time (ms)	syt1 KO neurons	KO (GFP)	2.9	0.4	11	Kruskal-Wallas	0.55	n/a
			syt1-FL	3.3	0.3	10			n/a
			syt1-C2A	2.5	0.3	10			n/a
			syt1-C2B	2.7	0.4	10			n/a
2H	τ Decay of post-train current (sec)	syt1 KO neurons	KO (GFP)	1.4	0.1	10	ANOVA; Sidak's Post-hoc	0.008	n/a
			syt1-FL	0.9	0.1	11			0.04 vs. KO
			syt1-C2A	1.4	0.1	10			0.97 vs. KO
			syt1-C2B	0.9	0.2	10			0.99 vs. syt1-FL
2J	Ratio of phasic current (2nd response over 1st)	syt1 KO neurons	KO (GFP)	1.6	0.2	10	Kruskal-Walls; Dunn's Post-hoc	<0.001	n/a
			syt1-FL	0.4	0.1	11			<0.001 vs. KO
			syt1-C2A	1.6	0.2	10			n/a
			syt1-C2B	1.7	0.3	10			<0.001 vs syt1-FL
2J	Ratio of phasic current (Steady-state response over 1st)	syt1 KO neurons	KO (GFP)	0.6	0.1	10	Kruskal-Walls; Dunn's Post-hoc	<0.001	n/a
			syt1-FL	0.2	0.1	11			0.012 vs. KO
			syt1-C2A	0.5	0.1	10			n/a
			syt1-C2B	2.9	0.8	10			0.039 vs KO; <0.001 vs. syt-FL
2K	Steady-state phasic amplitude (pA)	syt1 KO neurons	KO (GFP)	-89	20	10	Kruskal-Walls; Dunn's Post-hoc	0.002	n/a
			syt1-FL	-422	131	11			0.025 vs. KO
			syt1-C2A	-92	25	10			>0.99 vs. KO
			syt1-C2B	-305	74	10			0.81 vs. syt1-FL
3B	RRP (nC)	syt1 KO neurons	KO (GFP)	2.0	0.2	12	ANOVA; Dunnett's Post-hoc	0.0493	n/a
			syt1-FL	4.5	0.5	11			< 0.001 vs. KO
			syt1-C2A	2.4	0.6	10			0.54 vs. KO; 0.02 vs. syt1-FL
			syt1-C2B	4.4	0.8	10			0.006 vs. KO; 0.91 vs. syt1-FL
4B	IPSC Area (pC)	syt1 KO neurons	syt1-C2A & syt1-C2B	11	4	16	Kruskal-Wallas; Dunn's Post-hoc	<0.001	< 0.001 vs. syt1-FL; < 0.001 vs. syt1-C2A; 0.51 vs. syt1-C2B
4B	mIPSC Frequency (Hz)	syt1 KO neurons	syt1-C2A & syt1-C2B	0.6	0.2	14	ANOVA; Sidak's Post-hoc	<0.001	0.98 vs. syt1-FL; < 0.001 vs. syt1-C2A; 0.01 vs. syt1-C2B
4C	IPSC amplitude (pA)	WT mouse neurons (C57BL/6J)	GFP	-5003	680	11	ANOVA; Sidak's Post-hoc	<0.001	n/a
			syt1-FL	-3879	462	12			0.63 vs. GFP
			syt1-C2A	-4473	726	11			n/a
			syt1-C2B	-1488	453	11			<0.001 vs. GFP; 0.029 vs. syt1-FL
4D	mIPSC Frequency (Hz)	WT mouse neurons (C57BL/6J)	GFP	1.2	0.2	9	ANOVA; Sidak's Post-hoc	<0.001	n/a
			syt1-FL	1.3	0.2	11			0.99 vs. GFP
			syt1-C2A	1.8	0.3	13			n/a
			syt1-C2B	0.2	0.1	11			0.04 vs. GFP; 0.015 vs. sy1-FL
5C	mIPSC Frequency (Hz)	syt1 KO neurons	KO (GFP)	2.5	0.2	13	ANOVA; Sidak's Post-hoc	<0.001	n/a
			syt1-C2A	3.8	0.3	14			0.01 vs. KO
			syt1-C2A K189-192A	2.5	0.3	12			0.96 vs. KO; 0.01 vs. syt1-C2A
			syt1-C2A D230,232N	4.7	0.4	12			< 0.001 vs. KO; 0.1 vs. syt1-C2A
			syt1-C2A M173A, F234A	4.1	0.2	14			< 0.001 vs. KO; 0.47 vs. syt1-C2A
5G	IPSC Area (pC)	syt1 KO neurons	KO (GFP)	100	22	15	ANOVA; Sidak's Post-hoc	<0.001	n/a
			syt1-C2B	8	2	14			<0.001 vs. KO
			syt1-C2B R398,399Q	62	16	13			0.001 vs. KO; 0.001 vs. syt1-C2B
			syt1-C2B K326,327E	21	5	15			<0.001 vs. KO; 0.018 vs. syt1-C2B
			syt1-C2B V304A, I367A	3	1	15			<0.001 vs. KO; 0.016 vs. syt1-C2B
			syt1-C2B D363,365N	2	1	14			<0.001 vs. KO; 0.016 vs. syt1-C2B
5H	τ Charge Transfer (sec)	syt1 KO neurons	KO (GFP)	0.44	0.07	14	Kruskal-Wallas; Dunn's Post-hoc	<0.001	n/a
			syt1-C2B	0.16	0.02	8			0.008 vs. KO
			syt1-C2B R398,399Q	0.47	0.05	13			0.99 vs. KO; < 0.001 vs. syt1-C2B
			syt1-C2B K326,327E	0.51	0.09	13			0.99 vs. KO; 0.003 vs. syt1-C2B
5J	mIPSC Frequency (Hz)	syt1 KO neurons	KO (GFP)	2.6	0.3	15	ANOVA; Sidak's Post-hoc	<0.001	n/a
			syt1-C2B	0.2	0.1	15			< 0.001 vs. KO
			syt1-C2B R398,399Q	3.7	0.2	15			0.001 vs. KO; < 0.001 vs. syt1-C2B
			syt1-C2B K326,327E	1.3	0.2	16			< 0.001 vs. KO; < 0.001 vs. syt1-C2B
			syt1-C2B V304A, I367A	0.3	0.1	10			< 0.001 vs. KO; 0.99 vs. syt1 C2B
			syt1-C2B D363,365N	0.1	0.1	13			< 0.001 vs. KO; 0.99 vs. syt1 C2B
6C	Relative t-SNARE binding (normalized to C2AB in EGTA)	Recombinent Protein	C2AB - EGTA	1.00	n/a	4	Student's Paired T-Test	n/a	n/a
			C2AB - Ca2+	1.90	0.29	4			0.05 vs. C2AB - EGTA
			R398,399Q - EGTA	0.24	0.06	4			n/a
			R398,399Q - Ca2+	1.33	0.10	4			0.003 vs R398,399Q - EGTA
			K326,327E - EGTA	0.05	0.04	4			n/a
			K326,327E - Ca2+	0.88	0.14	4			0.005 vs. K326,327E - EGTA
6C	Relative t-SNARE binding (normalized to C2AB in EGTA)	Recombinent Protein	C2B - EGTA	0.41	0.18	4	Student's Paired T-Test	n/a	n/a
			C2B - Ca2+	1.21	0.30	4			0.025 vs. C2B - EGTA
			R398,399Q - EGTA	0.03	0.02	4			n/a
			R398,399Q - Ca2+	0.20	0.13	4			0.24 vs. R398,399Q - EGTA
			K326,327E - EGTA	0.04	0.03	4			n/a
			K326,327E - Ca2+	0.07	0.06	4			0.26 vs. K326,327E - EGTA

6F	Relative t-SNARE binding (normalized to C2AB in EGTA)	Recombinent Protein	tND, C2B- EGTA	1.13	0.21	3	Student's Paired T-Test	n/a	n/a
			tND, C2B - Ca2+	3.95	0.72	3			n/a
			transND, C2B - EGTA	1.0	n/a	4			n/a
			transND, C2B - Ca2+	3.55	0.59	4			0.022 vs. transND, C2B - EGTA
			transND, R398,399Q - EGTA	0.40	0.07	4			n/a
			transND, R398,399Q - Ca2+	1.97	0.39	4			0.018 vs. transND, R398,399Q - EGTA
			transND, K326,327E - EGTA	0.25	0.08	4			n/a
			transND, K326,327E - Ca2+	0.63	0.35	4			0.25 vs, transND, K326,327E - EGTA
7D	trans-SNARE complexes quanitified	Recombinent Protein	n/a				n/a	n/a	n/a
7D	trans-SNARE complexes bound to indicated protein (%)	Recombinent Protein	Only complexin	9.8	1.0	12	n/a	n/a	n/a
			Only Syt1-C2AB	5.7	1.0	12			n/a
			Both complexin and C2AB	5.3	0.5	12			n/a
7E	Frequency of both Complexin and syt1-C2AB being bound	Recombinent Protein	Calculated Random Chance	0.6	0.1	12	Student's Paired T-Test	n/a	n/a
			Observed	5.3	0.5	12			<0.001 vs Calculated Random Chance
7H	Complexin KD Efficency (% of Control)	WT mouse neurons (C57BL/6J)	n/a	89	2	8	n/a	n/a	n/a
7I	IPSC amplitude (pA)	WT mouse neurons (C57BL/6J)	Control	-4620	440	15	Student's T-Test	n/a	n/a
			Complexin I/II KD	-2480	490	14			0.003 vs. Control
7J	mIPSC Frequency (Hz)	WT mouse neurons (C57BL/6J)	Control	1.2	0.3	10	Student's T-Test	n/a	n/a
			Complexin I/II KD	1.0	0.2	10			0.58 vs. Control
7K	IPSC Area (nC)	syt1 KO neurons	KO (GFP), Control	219	43	15	Mann Whitney test	n/a	n/a
			KO (GFP), Cpx I/II KD	182	29	15			0.65 vs. KO Control
			syt1-FL, Control	919	144	15			n/a
			syt1-FL, Cpx I/II KD	390	63	15			< 0.001 vs. syt1-FL Control
			syt1-C2B, Control	17	5	13			n/a
			syt1-C2B, Cpx I/II KD	26	6	13			0.04 vs. syt1-C2B Control
7L	mIPSC Frequency (Hz)	syt1 KO neurons	KO (GFP), Control	6.9	0.5	13	Mann Whitney test	n/a	n/a
			KO (GFP), Cpx I/II KD	5.4	0.6	12			0.08 vs. KO Control
			syt1-FL, Control	2.0	0.2	16			n/a
			syt1-FL, Cpx I/II KD	2.3	0.2	16			0.17 vs. syt1-FL Control
			syt1-C2B, Control	0.2	0.1	9			n/a
			syt1-C2B, Cpx I/II KD	0.3	0.1	8			0.19 vs. syt1-C2B Control

Figure	Parameter	Experimental System	Condition (LV expression or Purified Protein)	Mean	SEM	N	Statistical Test	P-value (ANOVA)	P-Values (Indicated Comparisons)
S1D	Manders Colocalization (synaptophysin overlapping syt1)	syt1 KO neurons	KO (GFP)	0.05	0.01	6	Dunnett's Multiple Comparison Test	<0.001	n/a
			syt1-FL	0.55	0.08	9			<0.001 vs. KO
			syt1-C2A	0.29	0.02	6			0.02 vs. syt1-FL
			syt1-C2B	0.55	0.06	9			> 0.99 vs. syt1-FL
S2B	IPSC Area (pC)	Recombinent Protein	KO (GFP)	-116	29	11	Kruskal-Wallis; Dunn's Post-Hoc	<0.001	n/a
			syt1-C2B	-17	5	14			0.002 vs. KO
			syt1-C2B linker control	-8	3	12			<0.001 vs. KO; 0.87 vs. syt1-C2B
S2C	τ Charge Transfer (sec)	Recombinent Protein	KO (GFP)	0.42	0.06	9	Krusal-Wallis; Dunn's Post-Hoc	0.001	n/a
			syt1-C2B	0.14	0.03	11			0.003 vs. KO
			syt1-C2B linker control	0.19	0.04	10			0.009 vs. KO; 0.99 vs. syt1-C2B
S3C	Surface syt1 intensity (normalize to synaptophysin)	syt1 KO neurons	10x syt1-FL	185	4	9	ANOVA; Dunnett's Post-Hoc	<0.001	<0.001 vs. 1x
			1x syt1-FL	55	5	9			n/a
			0.1x syt1-FL	22	3	9			<0.001 vs. 1x
			0.01x syt1-FL	23	3	6			<0.001 vs. 1x
S3C	Internal syt1 intensity (normalize to synaptophysin)	syt1 KO neurons	10x syt1-FL	119	6	9	ANOVA; Test for linear trend	<0.001; <0.001 for linear trend	n/a
			1x syt1-FL	86	5	9			n/a
			0.1x syt1-FL	55	4	9			n/a
			0.01x syt1-FL	43	3	6			n/a
S4C	Pearson's Correlation Coefficient (syt1 with synaptophysin)	syt1 KO neurons	KO (GFP)	0.19	0.05	9	Student's T-Test	n/a	n/a
			syt1-FL	0.61	0.02	9			<0.001 vs. KO
S4C	Pearson's Correlation Coefficient (syt1 with synaptophysin)	syt1 KO neurons	syt1-C2A	0.43	0.02	6	Kruskal-Wallis	0.94	n/a
			syt1-C2A K189-192A	0.40	0.03	6			n/a
			syt1-C2A D230,232N	0.40	0.08	6			n/a
			syt1-C2A M173A, F234A	0.40	0.04	6			n/a
S4C	Pearson's Correlation Coefficient (syt1 with synaptophysin)	syt1 KO neurons	syt1-C2B	0.54	0.01	6	Kruskal-Wallis	0.07	n/a
			syt1-C2B R398,399Q	0.45	0.02	6			n/a
			syt1-C2B K326,327E	0.51	0.05	6			n/a
			syt1-C2B V304A, I367A	0.55	0.03	9			n/a
			syt1-C2B D363,365N	0.56	0.02	9			n/a
