# Supplementary material for: Synaptotagmin 1 clamps synaptic vesicle fusion in mammalian neurons independent of complexin
Source: Nat Commun. 2019 Sep 9;10:4076. doi: 10.1038/s41467-019-12015-w (PMC6733930; doi:10.1038/s41467-019-12015-w)
Supplement: Supplementary file 3 — Reporting Summary [file 41467_2019_12015_MOESM3_ESM.pdf]

nature research

Corresponding author(s): Edwin Chapman

Last updated by author(s): Aug 5, 2019

Reporting Summary

Nature Research wishes to improve the reproducibility of the work that we publish. This form provides structure for consistency and transparency in reporting. For further information on Nature Research policies, see Authors & Referees and the Editorial Policy Checklist .

Statistics

For all statistical analyses, confirm that the following items are present in the figure legend, table legend, main text, or Methods section.

n/a	Confirmed	
☐	☒	The exact sample size (n) for each experimental group/condition, given as a discrete number and unit of measurement
☐	☒	A statement on whether measurements were taken from distinct samples or whether the same sample was measured repeatedly
☐	☒	The statistical test(s) used AND whether they are one- or two-sided<br>*Only common tests should be described solely by name; describe more complex techniques in the Methods section.*
☐	☒	A description of all covariates tested
☐	☒	A description of any assumptions or corrections, such as tests of normality and adjustment for multiple comparisons
☐	☒	A full description of the statistical parameters including central tendency (e.g. means) or other basic estimates (e.g. regression coefficient) AND variation (e.g. standard deviation) or associated estimates of uncertainty (e.g. confidence intervals)
☐	☒	For null hypothesis testing, the test statistic (e.g. F, t, r) with confidence intervals, effect sizes, degrees of freedom and P value noted<br>*Give P values as exact values whenever suitable.*
☒	☐	For Bayesian analysis, information on the choice of priors and Markov chain Monte Carlo settings
☒	☐	For hierarchical and complex designs, identification of the appropriate level for tests and full reporting of outcomes
☐	☒	Estimates of effect sizes (e.g. Cohen's d, Pearson's r), indicating how they were calculated

Our web collection on statistics for biologists contains articles on many of the points above.

Software and code

Policy information about availability of computer code

Data collection	Commercial software used in this study are described in the Methods sections. These include: Digidata 1440A (Molecular Devices), Clampex 10 (Molecular Devices), Chimera (UCSF), Metamorph(Molecular Devices), and
Data analysis	Clampfit 10 (Molecular Devices), ImageJ, and GraphPad Prism (GraphPad Software) were used for data analysis, including statistical analysis.

For manuscripts utilizing custom algorithms or software that are central to the research but not yet described in published literature, software must be made available to editors/reviewers. We strongly encourage code deposition in a community repository (e.g. GitHub). See the Nature Research guidelines for submitting code & software for further information.

Data

Policy information about availability of data

All manuscripts must include a data availability statement. This statement should provide the following information, where applicable:

- Accession codes, unique identifiers, or web links for publicly available datasets
- A list of figures that have associated raw data
- A description of any restrictions on data availability

Data Availability Presented data can be found in the accompanying Supplementary Table 1; all other data available by request.

Field-specific reporting

Please select the one below that is the best fit for your research. If you are not sure, read the appropriate sections before making your selection.

☒ Life sciences ☐ Behavioural & social sciences ☐ Ecological, evolutionary & environmental sciences

Life sciences study design

All studies must disclose on these points even when the disclosure is negative.

Sample size	No statistical methods were used to predetermine sample size.
Data exclusions	Data were not excluded from analysis.
Replication	All experimental findings were reliably reproduced.
Randomization	Randomization was not performed for this study; a sufficient number of neurons was culture from each pup such that all conditions for a given experiment could be examined in parallel.
Blinding	Investigator was blinded when analyzing the frequency of spontaneous events. Other blinding was not performed.

Reporting for specific materials, systems and methods

We require information from authors about some types of materials, experimental systems and methods used in many studies. Here, indicate whether each material, system or method listed is relevant to your study. If you are not sure if a list item applies to your research, read the appropriate section before selecting a response.

Materials & experimental systems

n/a	Involved in the study	
☐	☒	Antibodies
☒	☐	Eukaryotic cell lines
☒	☐	Palaeontology
☐	☒	Animals and other organisms
☒	☐	Human research participants
☒	☐	Clinical data

Methods

n/a	Involved in the study	
☒	☐	ChIP-seq
☒	☐	Flow cytometry
☒	☐	MRI-based neuroimaging

Antibodies

Antibodies used	anti-synptotagmin-1: Invitrogen ASV48 and Cedarlane 105103(SY); anti-VCP: Abcam ab11433; anti-complexin I/II: Cedarlane 122022(SY); anti-GFP: Abcam ab290 and Abcam ab13970; and anti-synaptophysin: Cedarlane 101004(SY)
Validation	The validation statement can be found on the manufacturer's website. No signal was observed for syt1 antibodies target applied to syt1 KO material.

Animals and other organisms

Policy information about studies involving animals; ARRIVE guidelines recommended for reporting animal research

Laboratory animals	Neurons were cultured from syt1 KO mice (Jackson Laboratory; Stock #: 002478) at postnatal day 0-1. Sex was not determined.
Wild animals	This study did not involve wild animals.
Field-collected samples	This study did not involve samples collected from the field.
Ethics oversight	All procedures were in accordance with relevant ethical regulations, under the guidelines of the National Institutes of Health, and approved by the Animal Care and Use Committee at the University of Wisconsin – Madison.

Note that full information on the approval of the study protocol must also be provided in the manuscript.
