# Supplementary material for: Synaptotagmin 1 clamps synaptic vesicle fusion in mammalian neurons independent of complexin
Source: Nat Commun. 2019 Sep 9;10:4076. doi: 10.1038/s41467-019-12015-w (PMC6733930; doi:10.1038/s41467-019-12015-w)
Supplement: Supplementary file 2 — Peer Review [file 41467_2019_12015_MOESM2_ESM.pdf]

Reviewers' comments:

Reviewer #1 (Remarks to the Author):

This work presents a very detailed functional dissection of both C2 (C2A and C2B) domains of synaptotagmin (Syt1), one of the major players in synaptic transmission. Authors used a broad repertoire of very challenging techniques to grasp the molecular basis of the syt1 functions. The image of Syt1 that emerges from this study is that of a polyfunctional protein with each C2 domain playing different and, mainly not overlapping roles. The authors highlight the function of Syt1 as the ultimate clamp of the SNARE complex independent of complexin. This is an exciting and extremely well executed study. Conclusions are well supported and represent a major advance in the field. I strongly support publication with only minor suggestions.

Specific comments:

1. Electrophysiological measurements were based on recordings of inhibitory postsynaptic currents, while the majority of cells in cortical cultures are excitatory. Justification for this approach is needed.

2. The RRP is a very informative physiological measurement of the synapse. Although there is no consensus about how it should be measured, using high sucrose is a common but not the most physiologically relevant (or well understood) approach, and could potentially mix together the RRP and the recycling pool. Distinguishing between the two pools could be relevant given the roles described here for Syt1. The extrapolation method using analysis of cumulative release during steady-state depression (Schneggenburger et al., 1999) of the measured currents after a stimulation train is a plausible alternative that could be used even with the already recorded data.

3. Although not a central point of the ms, the conclusion that Syt1-C2A increases the mini frequency by promoting vesicle docking (Fig 2E) is interesting and can be further supported with EM experiments showing more vesicles tightly attached to the membrane. The same could be checked for R398,399Q mutant in syt1-C2B, to reveal if the same mechanism causes increase in the frequency of minis in Fig. 5I.

3. One point that can be discussed in more detail is the difference in the kinetics of release between syt1-C2A and syt1-C2B (Fig. 2D). Although the total amount of release was not recovered for either of the two ablated syt1, it's very interesting how the two components of release seem to be segregated by the two C2 domains.

4. Please clarify/expand justification for the use of molecular crowder to mimic complex environment in the synaptic boutons in fusion experiments (Fig 7). How the appropriate concentration of Ficoll 70 was determined?

Minor:
P 6. last par. References are not formatted correctly.
P 8. 3d par. “effects” should probably be “affects”.

Reviewer #2 (Remarks to the Author):

Neurons communicate each other via the controlled (or evoked) release of neurotransmitters in response to the action potential. Although tightly regulated spontaneous release (or leakage) happens in a low frequency. It has been previously shown that when Ca2+-sensor synaptotagmin 1 (syt1) is deleted the frequency of the spontaneous release increases significantly, suggesting that syt1 has the

capacity to contain these random events.
In this work, the authors narrows that the C2B domain of syt1 is solely responsible for the reduction of the spontaneous release by expressing truncation mutants syt1-C2B and syt1-C2A, each lacking one of two tandem C2 domains, in a syt1-deficient mouse model. Furthermore, the authors show that the specific interaction between the opposite side of the Ca2+-binding motifs and the SNARE complex is required for this function.
For long, it has been (perhaps wrongly) thought that complexins suppress spontaneous vesicle fusion by binding to the SNARE complex. The release of complexin by Ca2+-activated syt1 has been a popular mechanism by which vesicle fusion events are synchronized. However, in this work, the authors show that the deletion of complexins I and II does not affect the spontaneous release while reducing the amplitude of the evoked release.
Summing up, the authors propose a mechanistic model that syt1, not complexins, is the fusion clamp for the evoked release.
This is very well written paper with many valuable data that provide important insights into the molecular-level mechanism by which the fast evoked neurotransmitter release is brought about. This reviewer believes that the paper deserved a space in Nature Communications. However, there are some issues that the authors need to consider in moving forward with the publication.
1) Including the results presented in this work, there is now compelling evidence that the "complexin/syt1 switch model proposed by Sudhof et al. (2006 Cell) and by Rothman et al. (2006 Science) is most likely invalid. This mechanistic model is largely based on the premise that SNARE-induced membrane fusion is spontaneous and thus, it requires a fusion clamp. Now, provided that the complexin/syt1 switch model is not valid anymore I am wondering if the premise that the mechanism was based on is still valid. In other word, is the clamping of SNARE-dependent vesicle fusion necessary or required for evoked exocytosis? The experimental evidence that syt1-clamping is coupled with evoked vesicle fusion is lacking, and the authors need to provide the data that supports the proposed mechanism. Otherwise, Syt1 might be simply tightening the undesired leakage of neurotransmitters.
2) Even if syt1 is deleted the RRP vesicle pool is still nearly than half-filled (Figure 3). The results might suggest that SNARE complex formation may be a slow event although membrane fusion happens very fast once SNARE complex formation is complete. I am wondering these half-filled RRP vesicles are the primed vesicles or dead vesicles because they are not "clamped" by syt1.
3) Do the authors expect to see the enhancement of Ca2+-triggered membrane fusion if Ca2+ is made pre-bound to syt1 before v-liposomes are mixed with t-liposomes in their bulk in vitro assay? The results of this experiment might provide some answers to the question if "syt1-clamping" is required for Ca2+-triggered vesicle fusion. A similar experiment was already performed by Kim et al. (Frontiers 2017).

4) Coexistence of syt1 and complexin during Ca2+-triggered membrane fusion has been previously shown by Schaub et al (NSMB 2006).
5) On page 10, the authors state, "To the best of our knowledge, this is the first demonstration that trans-SNARE complexes and t-SNARE heterodimers may be equivalent targets for syt1." Such data has been already published (Fig. 1 of Lai et al. Scientific Reports 2015).
6) It is desirable if the authors verify the integrity of nanodisc sandwiches with some experimental means such as EM.

Reviewer #3 (Remarks to the Author):

This is a well crafted, highly interesting, and provocative paper which will likely be of wide interest to the neuroscience community. It establishes that 1) synaptotagmin 1 (syt1) clamps spontaneous release; 2) the introduction of the C2B domain alone into syt1 KO neurons clamps evoked release, and has a more profound effect on clamping spontaneous release than syt1 (suggesting that the C2B domain is responsible for the clamping action of syt1, and can act as a 'superclamp' when introduced

in isolation); 3) does so independent of complexin. Additionally, this paper provides evidence for the residues essential for the specific functions of the C2A and C2B domains with regards to modulate clamping/evoking release.

Crucially, however, some essential controls are missing that are required to validate the outcomes from the paper (listed below). Additionally, the authors fail to cite conflicting results (including from their previous work) to place their outcomes within the context of the field. These must be remedied before publication can be recommended.

Fig 1: This assay establishes that the internal fraction of syt1 variants colocalises with synaptophysin. Whilst this is an elegant assay to demonstrate that this internal fraction of syt1 is likely to be vesicular, this does not establish the degree to which syt1 localises to the internal (SV) vs plasma membrane fractions, which is also essential information to demonstrate that syt1 variants are targeted and trafficked correctly to vesicles (which has consequences for interpretation of all proceeding results). These assays are easy to perform as the syt1 variants are tagged with pHluorin. Authors should quench the surface fraction with acidic buffer and reveal total syt1 fluorescence with NH_4Cl to determine the extent to which syt1 variants are plasma membrane localised.

This is pertinent as the Authors have previously demonstrated (Yao Nat Neurosci 2011) that syt1-C2A and syt1-C2B only partially colocalises with synapsin (29%), as compared to full-length syt1 (71%), which differs considerably from the results from this new assay looking at the colocalisation of specifically the internal fraction (which would presumably select for the vesicular fraction anyway). These results together would suggest that there is indeed a very large component of syt1-C2A and syt1-C2B which is stranded on the plasma membrane, and which might explain, for example, the activity of syt1-C2B as a 'super-clamp'. It may just be that this variant is highly plasma-membrane localised, interacts with the fusion machinery on the plasma membrane, and therefore 'clamps' evoked and synchronous release by artificially poisoning the fusion machinery or blocking release sites, rather than being the true biological driver of clamping. This is relevant as well, as the authors showed in the same paper that localisation of syt1 to SVs (via syt1 C2AB fused to GAP43) isn't necessary to rescue exocytosis in syt1 KO neurons, demonstrating that plasma membrane localised syt1 can in fact interact with the fusion machinery.

Additionally, the authors state in their previous paper (Yao Nat Neurosci 2011) that 'Although the majority of boutons were devoid of the truncated mutant proteins, those boutons that contained the proteins showed robust immunofluorescence' suggesting that there is significant bouton-to-bouton differences in syt1-C2A and syt1-C2B localisation/expression. What bouton-to-bouton variability in expression/localisation do they see in their current system? Authors should perform quantitative immunolabelling (transfected vs untransfected in same field of view as a control) to look at how variable the bouton-specific expression of syt1 variants is, and if this differs between WT and syt1-C2A and syt1-C2B. This has implications on interpretation of results.

The ability of syt1-C2B to act as a super-clamp is also at odds with previous results from these authors (Yao Nat Neurosci 2011). In this paper, syt1-C2A and syt1-C2B (fused to pHluorin) were transfected into syt1 KO neurons, and elicited fusion (as evidenced by an increase in pHluorin fluorescence) when evoked with trains of stimuli. The authors should discuss these current results in the context of their previous findings.

The authors refer to a paper by Lee and Littleton (47) to introduce and support some claims. However, in this paper, they also see no difference in between the ability of C2A or C2B (alone) to evoke synchronous release or to contribute to spontaneous release (i.e. all display similarly increased mEJP frequency as syt-/-), arguing against the ability of the C2B domain to act as a super clamp. These conflicting results should be discussed.

p6 (related to fig 4): The suggestion that syt1-C2A and syt-C2B when coexpressed somehow regulate different pools/subsets of SVs lacks supporting evidence. Such a possibility would be supported if there is a very low targeting of syt1-C2A or syt1-C2B to boutons/synaptic vesicles, suggesting a stochastic response based on whichever syt1 variant dominates in that specific terminal. Is there a bimodal distribution in the data? More importantly, while coexpressing syt1-C2A and syt1-C2B is significantly different from expressing syt1-C2A or syt1-C2B alone for clamping spontaneous release, it is no different from syt1-FL. Is this not the more important and revealing finding? Suggests that as long as both domains are present (but not necessarily linked), there is normal clamping.

p8: Fig 7A-C: this is an elegant assay, but is qualitative. What does ‘many individual trans-SNARE complexes associated with both syt1C2AB and complexin’ mean? Most? It would be more powerful if this were quantified (eg % C2AB-syntaxin clusters also containing complexin, % complexin-syntaxin clusters also containing C2AB for each image) rather than stating in figure legend that ‘colocalisation was consistently observed over 12 images.’
Please provide n and p values for Fig 7D and E. Were any statistical analyses performed for this assay? Please indicate in figure legend whether this data is representative, and of how many individual assays.
p6, Fig 5: These results are very interesting, but are entirely dependent on the relative expression of each of these variants. Do these variants express equally as well as their relative C2A and C2B controls? Are they localised correctly to synaptic vesicles? The lack of effect of some variants (eg the D230,232N mutant) may simply be due to this variant not expressing/ localising to SVs correctly, thus recapitulating the KO phenotype.
p11: ‘over the course of evolution, the ability to clamp fusion may have migrated from complexin to the C2B domain of syt1’. There is no supporting evidence provided for this speculation. How conserved are, for example, mouse and drosophila synaptotagmin and complexin? How conserved is the C2B domain? Are there different isoforms of complexin and synaptotagmins in mammals vs invertebrates? If anything, other studies suggest that the two proteins interact to clamp spontaneous release in invertebrates (Jorquera + Littleton JT 2012 J Neurosci. Complexin null flies show increased frequency of minis, synaptotagmin null flies are no different from WT, and the complexin-dependent increase in minis is ablated when these flies are crossed with syt null flies).

Minor
p2: ‘All studies agree that loss of complexin impairs evoked SV fusion’. Please reference these studies.
p3: The RRP has previously been shown to be not altered in the absence of syt1 alone (Bacaj 2015 PLoS Biology), but only reduced when syt1 and syt7 are both depleted from neurons. These conflicting results should be referenced.
p6 (related to fig 4): ‘did not alter the amount of native protein (data not shown)’. Determined how? This result should be included in supplementary data.
Methods: details of mutants are missing from methods. Please provide additional details. It is not readily apparent that the mutations in fig 5 are present in the syt1-C2A or syt1-C2B constructs (rather than in full length syt1).
Please provide more details for transfection of neurons for localisation experiments.
Please provide details of what ‘n’ denotes for Fig 1 (localisation experiment). Are these also from multiple individual cultures?

Response to Reviewer Comments

Reviewer #1 (Remarks to the Author):

1. Electrophysiological measurements were based on recordings of inhibitory postsynaptic currents, while the majority of cells in cortical cultures are excitatory. Justification for this approach is needed.

In this study, we choose to use $GABA_A$-receptor mediated inhibitory postsynaptic currents as our model synapse for two reasons. First, a technical consideration: IPSCs, unlike excitatory currents, are not prone to the possibility of disynaptic currents require extra care and are difficult to avoid; they are especially problematic when kinetic analysis of the responses are an important goal. Additionally, it is nigh impossible to fully remove the influence of disynaptic currents from EPSCs during train stimulation, at least in cultured neurons. Second, a physiological consideration: our previous work showed that while syt1 influences the clamping of both mEPSCs and mIPSCs, only mIPSCs are promoted by syt1 in a Ca2+-dependent manner (Courtney et al., 2018 *Neuron*). So, measuring inhibitory currents seemed the most prudent approach to examining the overall function of syt1 in the synaptic vesicle cycle. For both of these reasons, we choose to use inhibitory synapses as our model to study the molecular function of syt1.

We have added text to the Methods section of the manuscript to address this concern:

"$GABA_A$ receptor mediated inhibitory postsynaptic currents (IPSCs) were examined to avoid disynaptic currents and because syt1 promotes inhibitory, but not excitatory, spontaneous events [Courtney 2018]."

2. The RRP is a very informative physiological measurement of the synapse. Although there is no consensus about how it should be measured, using high sucrose is a common but not the most physiologically relevant (or well understood) approach, and could potentially mix together the RRP and the recycling pool. Distinguishing between the two pools could be relevant given the roles described here for Syt1. The extrapolation method using analysis of cumulative release during steady-state depression (Schneggenburger et al., 1999) of the measured currents after a stimulation train is a plausible alternative that could be used even with the already recorded data.

We thank the reviewer for this comment, and agree that the mechanism that underlies the sucrose response is not fully understood. However, we note that sucrose application and AP-dependent stimulation have been shown to share the same pool of vesicles by Rosenmund and Stevens in Neuron (1996). Additionally, our experiments quantified only the 'acute,' phasic current with sucrose application (which is prominent for only the first couple of seconds in the 15 second application) while excluding the 'steady-state,' tonic current – this helps to separate the RRP from the recycling pool and recycling rate.

While it is common to measure the RRP using train stimulation data in many cases (and we have done so in other papers from our lab), we do not believe that this approach is appropriate in this specific case: when analyzing syt1 KO neurons. Because evoked release in syt1 KOs is asynchronous, a tonic current quickly builds up that significantly complicates measuring phasic release – it is this phasic current that is quantified for RRP calculations. The only way to avoid this tonic current is to stimulate either with single APs or very slow frequency trains. This is an issue because the speed of the stimulus trains directly correlates to the accuracy of the RRP measurement (slow trains are more inaccurate due to the increased influence of replenishment). In line with this,

we cannot find any case in the literature where train-stimulation protocols have been used to measure the RRP of syt1 KO neurons; all studies that we are familiar with – from multiple labs – use a sucrose-based RRP approach. So, with respect, we strongly believe that the sucrose approach is the correct method specifically when studying syt1 KO neurons, and we have opted to not analyze the train data to try to estimate the RRP.

3. Although not a central point of the ms, the conclusion that Syt1-C2A increases the mini frequency by promoting vesicle docking (Fig 2E) is interesting and can be further supported with EM experiments showing more vesicles tightly attached to the membrane. The same could be checked for R398,399Q mutant in syt1-C2B, to reveal if the same mechanism causes increase in the frequency of minis in Fig. 5I.

We agree with the reviewer that EM images of the docked vesicle pool in syt1 KO neurons expressing these domain-deletion mutants would be very interesting. Indeed, we began collaborating with another investigator to generate these images some time ago. However, these experiments are taking much longer than anticipated, and we have run into a number of unexpected hurdles. So, while we will absolutely continue this line of study, we believe that these data would be better suited to a follow-up report based on both the expected timeline for data acquisition (will take roughly a year) and, as the reviewer notes, the tangential relation to the objectives of the current manuscript.

3. One point that can be discussed in more detail is the difference in the kinetics of release between syt1-C2A and syt1-C2B (Fig. 2D). Although the total amount of release was not recovered for either of the two ablated syt1, it's very interesting how the two components of release seem to be segregated by the two C2 domains.

This appears to be a misunderstanding, and we apologize for not being clearer in our manuscript. While expression of syt1-C2A did increase the rate of miniature events, there was no significant difference in evoked release between syt1 KO neurons and those neurons expressing syt1-C2A. Thus, the kinetics of release in both cases (syt1-C2A and syt1 KO) are likely to be mediated by some compensatory Ca^{2+}-sensor.

We edited the Results text of the manuscript to more clearly make this distinction (new text in red):

"In neurons expressing syt1-C2A, evoked IPSCs were no different than those recorded from KO neurons (amplitude: -238 ± 26 pA, n = 15 responses with 1 failure, p = 0.40 vs. KO [amplitude]; total charge: 122 ± 18 pC, n = 16, p = 0.09 vs. KO; charge transfer: single exponential fit, τ = 0.61 ± 0.1 sec, n = 15, p = 0.94 vs. KO; Fig.2A-D), so it is unlikely that the syt1-C2A construct contributed to the regulation of evoked fusion."

4. Please clarify/expand justification for the use of molecular crowder to mimic complex environment in the synaptic boutons in fusion experiments (Fig 7). How the appropriate concentration of Ficoll 70 was determined?

We thank the reviewer for this comment, and have expanded our justification in the revised manuscript. Ficoll 70 has previously been used as a molecular crowder to mimic the complex, and crowded, environment in cells [Homouz et al., 2008 PNAS; Yu et al., 2015 JACS]. Of note, the work from Yu et al. (2015) highlighted the importance of these crowders specifically when studying the function of SNAREs (and, in their case, regulation by SM proteins). So, we used the same concentration as these prior studies, and we have now included these references in the manuscript.

Minor:
P 6. last par. References are not formatted correctly.

Thank you very much, this error has been corrected.

P 8. 3d par. "effects" should probably be "affects".

Thank you. We agree, and this has been corrected.

Reviewer #2 (Remarks to the Author):

1) Including the results presented in this work, there is now compelling evidence that the "complexin/syt1 switch model proposed by Sudhof et al. (2006 Cell) and by Rothman et al. (2006 Science) is most likely invalid. This mechanistic model is largely based on the premise that SNARE-induced membrane fusion is spontaneous and thus, it requires a fusion clamp. Now, provided that the complexin/syt1 switch model is not valid anymore I am wondering if the premise that the mechanism was based on is still valid. In other word, is the clamping of SNARE-dependent vesicle fusion necessary or required for evoked exocytosis? The experimental evidence that syt1-clamping is coupled with evoked vesicle fusion is lacking, and the authors need to provide the data that supports the proposed mechanism. Otherwise, Syt1 might be simply tightening the undesired leakage of neurotransmitters.

This is an interesting idea. We do believe that the underlying mechanism (i.e. that vesicles are first clamped and then trigger to fuse) is still valid – and that only the identity of the clamp proposed by Sudhof and Rothman was, in our opinion, incorrect (specifically in mammalian neurons). Indeed, the greatly increased rate of spontaneous events in syt1 KO neurons is consistent with the "*premise that SNARE-induced membrane fusion is spontaneous and thus, it requires a fusion clamp"* – loss of the clamp (syt1) increases spontaneous SNARE-mediated fusion (i.e. miniature events).

We believe that our data argues against a model by which syt1 simply tightens undesired leakage of neurotransmitters. Specifically, we note that expressing syt1-C2B did not merely clamp spontaneous and asynchronous release in syt1 KO neurons – it also resulted in the appearance of a synchronous component of release that was completely absent in KO neurons (Fig. 2D). Additionally, the residual release in syt1 KO neurons expressing syt1-C2B appears to be driven by C2B – both Ca^{2+} binding and membrane penetration by C2B was absolutely required for release in these conditions (Fig. 5). This argues against a passive role of C2B in simply clamping undesired leakage and suggests that the ability to both clamp and drive release resides in the same C2 domain (C2B). Finally, all mutations that we examined which broke the clamping function of C2B also broke the synchronicity of release (R398,399Q and K326,327E). This is consistent with the two events being part of the same mechanistic pathway and supports the coupling of syt1-mediated clamping and fusion.

Together, we believe that data which support this hypothesis are already present in the manuscript, and we unsure what additional data or experiments could be included. We have altered in the Discussion section of the manuscript to more clearly explain our conclusion as indicated below.

"While syt1-C2B strongly clamped evoked fusion in syt1 KO neurons, a small amount of evoked release was still observed. Interestingly, this residual release was synchronous and matched the fast component of evoked release in neurons expressing full-length syt1; this fast kinetic component was completely absent in syt1 KO neurons (Fig. 2). Based on mutagenesis (Fig. 5), the low levels of synchronous release results from the Ca^{2+}-binding and membrane penetration activity of C2B; this mechanism is similar to the canonical activity of the full length protein[37,50]. Thus, C2B appears to have multiple functions and is crucial for first arresting SVs and then facilitating synchronous fusion."

2) Even if syt1 is deleted the RRP vesicle pool is still nearly than half-filled (Figure 3). The results might suggest that SNARE complex formation may be a slow event although membrane fusion happens very fast once SNARE complex formation is complete. I am wondering these

half-filled RRP vesicles are the primed vesicles or dead vesicles because they are not "clamped" by syt1.

We apologize for not fully understanding this comment, so we hope the answer below will suffice.

We first note that EM tomographs from WT and syt1 KO neurons have found a decrease in the number of docked vesicles (Reist et al., 1998 *J Neurosci*; Liu et al., 2009 *J Neurosci*; Change et al., 2018 *Nat Neuro*) that is similar in magnitude to the decrease in RRP. This has two implications: 1) it is unlikely that the "half-filled" phenotype of the functionally measured RRP is due to dead vesicles and 2) the remaining docked vesicles in syt1 KO neurons must be 'primed' in some fashion since they can be triggered to release (i.e. contribute to the functionally measured RRP). However, if the C2B domain of syt1 contributes to the priming reaction, then the docked vesicles in syt1 KO neurons must have undergone some form of a compensatory priming pathway. This alteration in the docking/priming pathway is consistent with (and perhaps causative for) the decreased number of docked vesicles and the shift in release kinetics from synchronous to asynchronous seen in syt1 KO neurons. Interestingly, recently published work from the Regehr lab (Turecek et al., 2019 *Neuron*) found some CNS synapses that natively lack a fast synaptotagmin (i.e. syt1 or syt2). In these rather odd synapses, it appears as though syt7 can cover some of the functions normally attributed to syt1; i.e. KO of syt7 in these neurons increased asynchronous and spontaneous release similar to the phenotype of syt1 KO (in neurons that do natively express syt1). So, it is possible that other mechanisms, such as other Ca^{2+} sensors, can partially compensate for the loss of syt1 and assist docking/priming vesicles in syt1 KO neurons.

3) Do the authors expect to see the enhancement of Ca2+-triggered membrane fusion if Ca2+ is made pre-bound to syt1 before v-liposomes are mixed with t-liposomes in their bulk in vitro assay? The results of this experiment might provide some answers to the question if "syt1-clamping" is required for Ca2+-triggered vesicle fusion. A similar experiment was already performed by Kim et al. (Frontiers 2017).

In the bulk fusion assay, previous studies from our lab have shown that "syt1-clamping" in the absence of Ca^{2+} is crucial to increasing subsequent Ca^{2+}-triggered vesicle fusion (Chicka et al., 2008 NSMB). In a subsequent study form our lab (Wang et al., 2011 JCB), we found that when full length syt1, reconstituted into v-SNARE vesicles, must be preincubated with t-SNARE vesicles for a certain length of time in order for Ca2+ triggered fusion to occur at all. If Ca2+ is added prior to this preincubation, syt1 will interact with the cis (i.e. v-SNARE vesicle) membrane and will therefore not engage the target membrane to trigger fusion. So, we do not observe an enhancement if Ca^{2+} is pre-bound to syt1, rather, we see a fusion failure. Our group has shown the PIP2 plays a key role in 'steering' syt1 – prior to Ca^{2+}-binding - toward the t-SNARE membrane as part of an essential step for fusion (see also Bai et al. (2004) NSMB).

In reference to the Kim et al. (2017) study, the major effect of the pre-binding of Ca^{2+} in the membrane fusion assay (Fig. 2D vs. Fig. 3F-G) appeared to be a hastening in the pre-fusion docking and priming step – the rate of docking is greatly increased and the delay between docking and fusion (which likely includes a priming ration) is reduced. Importantly, and distinct from our above referenced data, the lipid composition of the vesicles in Kim et al. contained only 5% PS (our studies use >15%) and no PIP2. As a result, syt1●Ca^{2+} would tend to avoid binding the vesicle membrane (this interaction requires acidic phospholipids and greatly diminishes when PS is below 10%), and would greatly prefer to bind to the supported bilayer (which contained 15% PS and PIP2 – an even more important lipid for directing syt1 binding). Hence, under these lipid conditions,

the major effect of Ca^{2+} pre-incubation is on the docking/priming reaction. In cells, this reaction would be catalyzed by a multitude of additional factors such as Sec/Munc proteins, and would occur before the Ca^{2+}-trigger and the formation of syt1●Ca^{2+}. Again, in our hands, allowing the t- and v-SNARE bearing liposomes to pre-incubate for an extended period of time, in the absence of Ca^{2+}, allows for docking/priming to occur and is critical to reconstituting fast syt1●Ca^{2+} driven fusion.

4) Coexistence of syt1 and complexin during Ca2+-triggered membrane fusion has been previously shown by Schaub et al (NSMB 2006).

We thank the reviewer for pointing out this missed reference. Schaub et al. (2006) *NSMB* did suggest concurrent binding of syt1 and complexin with ternary *cis*-SNAREs (liposomes bearing t-SNARE heterodimers that were associated with the soluble domain of VAMP2) in the presence of Ca^{2+} and saturating amounts of complexin using bulk pull-down experiments. We have now included this reference in the revised manuscript.

"The key finding was that many of the individual *trans*-SNARE complexes associated with both syt1-C2AB and complexin (5.3 ± 0.5%; 12 fields of view; 806 total *trans*-SNARE complexes; Fig. 7D), consistent with previously reported ensemble experiments that examined ternary *cis*-SNARE complexes [Schaub 2006][Chicka 2009]."

5) On page 10, the authors state, "To the best of our knowledge, this is the first demonstration that trans-SNARE complexes and t-SNARE heterodimers may be equivalent targets for syt1." Such data has been already published (Fig. 1 of Lai et al. Scientific Reports 2015).

We thank the reviewer for noting this, and apologize for missing this reference.

Lai et al. (2015) examined the ternary *cis*-SNARE complex, not the *trans*-SNARE complexes. Their ternary was formed by SNAP-25 and truncated forms of syntaxin 1A (191-266) and VAMP2 (1-96); these truncations removed the transmembrane domains so the SNAREs could be studied in solution. Thus, Lai et al. did not examine *trans*-SNARE complexes utilizing full-length proteins embedded in opposing membranes. We also note that Lai et al. nicely confirmed an earlier study from our lab (Bai et al. (2004) Neuron) showing that the cytoplasmic domain of syt1 recognizes isolated t-SNAREs, t-SNARE heterodimers, and ternary SNARE complexes equally well. To address this issue, we have made the following addition to the manuscript to note the work of Lai et al.:

"To the best of our knowledge, this is the first demonstration that *trans*-SNARE complexes and t-SNARE heterodimers may be equivalent targets for syt1. This result is consistent with previous results examining truncated ternary *cis*-SNARE complexes [Bai 2004][Lai 2015]."

6) It is desirable if the authors verify the integrity of nanodisc sandwiches with some experimental means such as EM.

This is an important consideration for the interpretation of our single molecule and pull-down experiments. The integrity of trans-SNARE nanodisc (ND) sandwiches have been examined in previous studies and were shown to be stable complexes via single molecule imaging experiments (Shin et al., 2014 Biochem J). In our study, trans-SNAREs were reconstituted using NDs, and were purified using sucrose gradients to isolate only trans-complexes that contain both v- and t-SNARE NDs, as described by Shin et al. (2014). This is an established method, so we do not anticipated any issues

regarding the integrity of the ND complexes. Moreover, we have performed sm-FRET experiments to study the conformational state of trans-SNARE complexes formed between two NDs (as part of another study currently under consideration) and found that that trans-SNARE NDs indeed bear partially zippered SNARE complexes (we can provide these data, for the referee, if deemed necessary).

Reviewer #3 (Remarks to the Author):

Fig 1: This assay establishes that the internal fraction of syt1 variants colocalises with synaptophysin. Whilst this is an elegant assay to demonstrate that this internal fraction of syt1 is likely to be vesicular, this does not establish the degree to which syt1 localises to the internal (SV) vs plasma membrane fractions, which is also essential information to demonstrate that syt1 variants are targeted and trafficked correctly to vesicles (which has consequences for interpretation of all proceeding results). These assays are easy to perform as the syt1 variants are tagged with pHluorin. Authors should quench the surface fraction with acidic buffer and reveal total syt1 fluorescence with NH4Cl to determine the extent to which syt1 variants are plasma membrane localised. This is pertinent as the Authors have previously demonstrated (Yao Nat Neurosci 2011) that syt1-C2A and syt1-C2B only partially colocalises with synapsin (29%), as compared to full-length syt1 (71%), which differs considerably from the results from this new assay looking at the colocalisation of specifically the internal fraction (which would presumably select for the vesicular fraction anyway). These results together would suggest that there is indeed a very large component of syt1-C2A and syt1-C2B which is stranded on the plasma membrane, and which might explain, for example, the activity of syt1-C2B as a 'super-clamp'. It may just be that this variant is highly plasma-membrane localised, interacts with the fusion machinery on the plasma membrane, and therefore 'clamps' evoked and synchronous release by artificially poisoning the fusion machinery or blocking release sites, rather than being the true biological driver of clamping. This is relevant as well, as the authors showed in the same paper that localisation of syt1 to SVs (via syt1 C2AB fused to GAP43) isn't necessary to rescue exocytosis in syt1 KO neurons, demonstrating that plasma membrane localised syt1 can in fact interact with the fusion machinery. Additionally, the authors state in their previous paper (Yao Nat Neurosci 2011) that 'Although the majority of boutons were devoid of the truncated mutant proteins, those boutons that contained the proteins showed robust immunofluorescence' suggesting that there is significant bouton-to-bouton differences in syt1-C2A and syt1-C2B localisation/expression. What bouton-to-bouton variability in expression/localisation do they see in their current system? Authors should perform quantitative immunolabelling (transfected vs untransfected in same field of view as a control) to look at how variable the bouton-specific expression of syt1 variants is, and if this differs between WT and syt1-C2A and syt1-C2B. This has implications on interpretation of results. The ability of syt1-C2B to act as a super-clamp is also at odds with previous results from these authors (Yao Nat Neurosci 2011). In this paper, syt1-C2A and syt1-C2B (fused to pHluorin) were transfected into syt1 KO neurons, and elicited fusion (as evidenced by an increase in pHluorin fluorescence) when evoked with trains of stimuli. The authors should discuss these current results in the context of their previous findings. The authors refer to a paper by Lee and Littleton (47) to introduce and support some claims. However, in this paper, they also see no difference in between the ability of C2A or C2B (alone) to evoke synchronous release or to contribute to spontaneous release (i.e. all display similarly increased mEJP frequency as syt-/-), arguing against the ability of the C2B domain to act as a super clamp. These conflicting results should be discussed.

We thank the reviewer for this very insightful comment. We believe that this comment touches on five issues, and we would like to break down our response accordingly.

A) "*It may just be that [syt1-C2B] is highly plasma-membrane localised, interacts with the fusion machinery on the plasma membrane, and therefore 'clamps' evoked and synchronous release by artificially poisoning the fusion machinery or blocking release sites, rather than being the true biological driver of clamping.*"

Whether the high surface fraction is the cause of the super clamping phenotype is a crucial, and very insightful, question – we thank the referee. In short, new experiments

now demonstrate that the surface fraction of syt1 does not contribute to the clamping of exocytosis.

We have examined this issue in a new supplemental figure that demonstrates that over-expression of syt1-FL results in a dramatic increase in the fraction of syt1 on the plasma membrane, in both absolute and relative (to the internal fraction) terms. This is notable because over-expressing syt1 in otherwise WT neurons (corresponding to the high expression paradigm in the new supplemental figure) does not result in a super clamped (or any other observed) phenotype: both the rate of miniature events and the size and shape of the single stim evoked response were unchanged with over-expression of syt1-FL in WT neurons (Fig. 4C and D). Thus, a higher PM localization cannot explain a super clamped phenotype.

This new result – that the surface fraction does not appear to be involved in clamping – is consistent with other data in our manuscript. First, it is difficult to envision how syt1-C2B would poison the fusion machinery or block release sites in the PM without significantly reducing the RRP; we observed that the RRP in syt1-C2B expressing neurons was the same as in neurons expressing syt1-FL (Fig. 3). Second, the synchronous kinetics of residual evoked release in C2B-expressing neurons strongly suggest that fusion is being actively driven by syt1-C2B, as opposed to being driven by the compensatory sensor in syt1 KOs in combination with incomplete clamping by C2B. This residual release follows established rules for release mediated by full length syt1: it requires both Ca^{2+}-binding and membrane penetration (Fig. 5). So, we believe it very likely that syt1-C2B is acting through the canonical mechanism of syt1-mediated fusion.

The propensity for syt1 to utilize the PM as a reservoir for over-expressed protein makes the proposed pHluorin experiment quite difficult to perform and interpret. More importantly, we feel that the above approach addressed the root of this concern by demonstrating that the surface fraction of syt1 is unlikely to regulate synaptic vesicle clamping or fusion. Given these new data and conclusion, we argue that the proposed pHluorin experiments will not provide further insights, so this experiment was not conducted.

The following text has been included in the Results section to address this concern, along with a new Supplemental Figure 3:

"Importantly, overexpression of syt1-FL in WT neurons did not alter the single stimulation evoked response (Fig. 4C) or the frequency of miniature events (Fig. 4D). Separate localization experiments in syt1 KO neurons, utilizing the pH-syt1 construct (Fig. 1), show a drastic increase in plasma membrane localized fraction of syt1 when overexpressed (Supp. Fig. 3B and C). The lack of any phenotype from syt1 overexpression, despite the greatly increased surface fraction, argues against a role for plasma membrane localized syt1 in clamping SV fusion."

B) *"The Authors have previously demonstrated (Yao Nat Neurosci 2011) that syt1-C2A and syt1-C2B only partially colocalises with synapsin (29%), as compared to full-length syt1 (71%), which differs considerably from the results from this new assay looking at the colocalisation of specifically the internal fraction (which would presumably select for the vesicular fraction anyway) … What bouton-to-bouton variability in expression/localisation do they see in their current system?"*

This is an important consideration, and we thank the reviewer for raising this issue. To determine the relative bouton-to-bouton variability of our constructs, we used a virus-based approach. In our hands, lentivirus is more suited for controlling for expression

levels, which is critical to accurately compare the localization of the three constructs given the propensity for overexpressed syt1 to fill the PM (see point A, above). Furthermore, use of lentivirus allows for a more straightforward comparison with our electrophysiological experiments.

In these experiments, we quantified the overlap between synaptic puncta (marked by synaptophysin) and our syt1-FL, syt1-C2A, and syt1-C2B constructs using a Mander's overlap analysis. Our results do, in part, contradict the earlier Yao et al. study. We find that syt1-FL and syt1-C2B equally overlap with synaptic punta; i.e. syt1-FL and syt1-C2B appear to have the same bouton-to-bouton variability. This is consistent with our functional data as it would be hard to rationalize how syt1-C2B could shut down nearly all spontaneous events if it was only present at a subset of boutons. In line with the Yao et al. report, syt1-C2A had significantly worse overlap with synaptophysin. This indicates that sorting to boutons was impaired, but not ablated, by the loss of C2B. We have included this observation in the revised manuscript. Given that the primary findings of the current study focus on C2B, we do not believe that these data impact our conclusions. These data is presented as a new panel D in Supplemental Figure 1 with changes to the accompanying Results text (quoted below).

As to why some of our current results differ from the Yao study, we offer two potential explanations. First, the constructs in Yao et al. were all tagged with pHluorin on the N-terminus while this new Supplemental analysis localizes untagged proteins; the N-terminal tag may have somehow impacted the bouton-to-bouton variability. Second, and perhaps more importantly, we have completely updated how we generate lentivirus in our laboratory since the publication of Yao et al. in 2011. This update includes all new reagents, transfer plasmids, and harvesting protocols. With this new virus system, we have greater confidence in our ability to carefully control expression levels while maintaining complete coverage (which we monitor and quantify by an IRES-expression GFP). So, the difference in the previous and current studies may have arisen from better infection protocols.

"Next, we examined how efficiently these domain-deletion constructs targeted to boutons. Each construct was virally expressed (minus the pHluorin tag) in syt1 KO neurons at similar levels (Supp. Fig. 1B and C), and colocalization experiments examined the overlap of each construct with synaptophysin. Syt1-C2B accumulated in synaptic boutons as well as syt1-FL (Mander's coefficient, synaptophysin overlapping with syt1; syt1-FL: 0.55 ± 0.08, $n = 9$; syt1-C2B: 0.55 ± 0.06, $n = 9$; $p > 0.99$, Dunnett's multiple comparison's test; Supp. Fig. 1D). However, syt1-C2A had a significantly lower overlap with synaptophysin (0.29 ± 0.02, $n = 6$; $p = 0.02$ vs. syt1-FL; Supp. Fig. 1D), indicating that sorting was impaired. Thus, C2B is not absolutely essential for targeting syt1 to at least some boutons. However, this domain ensures that syt1 is targeted to virtually all synapses."

C) *This is relevant as well, as the authors showed in the same paper that localisation of syt1 to SVs (via syt1 C2AB fused to GAP43) isn't necessary to rescue exocytosis in syt1 KO neurons, demonstrating that plasma membrane localised syt1 can in fact interact with the fusion machinery.*

We thank the referee for this question. In Yao et al. we stated: "… we cannot exclude the possibility that the low levels of GAP43-C2AB on SVs (Fig. 1e) were sufficient to regulate normal rates of release but not normal rates of vesicle retrieval". We have

since learned, from Volker Haucke, that his laboratory was able to detect some level of this fusion protein on SV clusters by immune-gold EM, consistent with the fractionation experiments in Yao et al. (2011). Moreover, another lab that we interact with has a study in review in which they claim a single copy of syt1 can drive evoked fusion. So, at present, it is unclear which pool of the GAP43-C2AB rescued synchronous release in syt1 KO neurons in Yao et al. (2011). However, given points A and B above (that syt1-C2B reaches the same fraction of boutons as syt-FL, and that loading the PM with syt1-FL has no measured phenotype), this question does not impact the experiments, interpretation, or conclusions in our current manuscript.

D) *The ability of syt1-C2B to act as a super-clamp is also at odds with previous results from these authors (Yao Nat Neurosci 2011). In this paper, syt1-C2A and syt1-C2B (fused to pHluorin) were transfected into syt1 KO neurons, and elicited fusion (as evidenced by an increase in pHluorin fluorescence) when evoked with trains of stimuli. The authors should discuss these current results in the context of their previous findings.*

This appears to be a misunderstanding. In the pHluorin experiments presented in Yao et al., it is important to note that: 1) only normalized traces were presented, and 2) no differences were observed in the rising phase kinetics of the normalized pHluorin trace under any condition, including between *WT neurons and syt1 KO neurons* (Fig. 6D). This imaging approach - at least in our hands - simply does not have the temporal resolution needed to reveal changes in the rate of release in syt1 KO neurons. Thus, at least in those kinds of experiments, the rising phase kinetics of the pHluorin response to train stimulation could not provide information regarding the function of syt1 in synchronizing fusion. Instead, the Yao et al. data were meant to emphasize the contribution of each C2 domain in the process of endocytosis. Endocytosis is relatively slow, and is thus amenable to analysis using pHluorins. In short, we do not believe that our current work and the work of Yao et al. 2011 are at odds – the pHluorin experiments could not, and were not meant to, provide any information regarding the role of C2A or C2B in exocytosis.

E) *The authors refer to a paper by Lee and Littleton (47) to introduce and support some claims. However, in this paper, they also see no difference in between the ability of C2A or C2B (alone) to evoke synchronous release or to contribute to spontaneous release (i.e. all display similarly increased mEJP frequency as syt-/-), arguing against the ability of the C2B domain to act as a super clamp. These conflicting results should be discussed.*

We thank the reviewer for pointing out this inconsistency. We have now included this point in the Discussion text (quoted below). We believe that, similar to the role of complexin, this may simply reflect a difference in the underlying biology of mammals and flies.

"The most striking observation in the current study was that syt1-C2B serves as a powerful clamp that inhibits all measured forms of SV release (Fig. 2). This appears to be specific for mammalian neurons as clamping was not observed in similar experiments in *Drosophila* preparations [Lee and Littleton 2013]."

and

"We find it intriguing that complexin clearly functions to limit fusion in invertebrate neurons[57,58] and speculate that, over the course of evolution, the ability to clamp fusion

may have migrated from complexin to the C2B domain of syt1. Indeed, the function of complexin is hypothesized to have evolved between invertebrates and mammals[15] and the C2B domain of syt1 does not appear to clamp fusion at the *Drosophila* neuromuscular junction [Lee and Littleton 2013].”

p6 (related to fig 4): The suggestion that syt1-C2A and syt-C2B when coexpressed somehow regulate different pools/subsets of SVs lacks supporting evidence. Such a possibility would be supported if there is a very low targeting of syt1-C2A or syt1-C2B to boutons/synaptic vesicles, suggesting a stochastic response based on whichever syt1 variant dominates in that specific terminal. Is there a bimodal distribution in the data? More importantly, while coexpressing syt1-C2A and syt1-C2B is significantly different from expressing syt1-C2A or syt1-C2B alone for clamping spontaneous release, it is no different from syt1-FL. Is this not the more important and revealing finding? Suggests that as long as both domains are present (but not necessarily linked), there is normal clamping.

We thank the referee for this question. We believe that the data support our original interpretation, and apologize for not fully explaining our reasoning in the original submission. A key result that sheds light on the interpretation of these data is that overexpression of syt1-C2B alone, in otherwise WT neurons, also clamps both evoked and spontaneous release (Figure A and B). If all that was required was for both domains to be present (but not necessarily linked), then there should have been no effect of expressing C2B in these WT neurons.

We do not observe a bimodal distribution of the data; however, we argue that a bimodal distribution would not to be expected. We quantified the whole-neuron rates of spontaneous events, but the determinant of C2A vs. C2B mediated regulation (i.e. to fuse or be clamped) was likely to occur on the level of individual synaptic vesicles, or even individual fusion complexes. To see a bimodal distribution, we would need to somehow measure fate of individual vesicles (or, potentially, even individual fusion complexes).

We have modified the Results text to better reflect our line of reasoning:

“This suggests that syt1-C2B acts in a dominant negative manner and that the WT protein is unable to rescue or overcome the function of this deletion mutant. Furthermore, this finding provides additional evidence that the C2-domains must be linked in tandem for full function during spontaneous and evoked release.”

p8: Fig 7A-C: this is an elegant assay, but is qualitative. What does ‘many individual trans-SNARE complexes associated with both syt1C2AB and complexin’ mean? Most? It would be more powerful if this were quantified (eg % C2AB-syntaxin clusters also containing complexin, % complexin-syntaxin clusters also containing C2AB for each image) rather than stating in figure legend that ‘colocalisation was consistently observed over 12 images.’
Please provide n and p values for Fig 7D and E. Were any statistical analyses performed for this assay? Please indicate in figure legend whether this data is representative, and of how many individual assays.

We thank the reviewer for this suggestion; conducting this detailed analysis has yielded new and exciting insights. At the protein concentrations used in our experiments, we observed that ~6% of *trans*-SNARE complexes bound both syt1-C2AB and complexin, while ~10% and ~5% bound either complexin or syt1-C2AB alone, respectively. This data came from quantifying 806 total complexes spread over 12 experiments from at least three independent preparations.

The interesting new insight is as follows: given the observed individual frequencies of binding, random chance would predict that the SNARE-syt1C2AB-complexin complex should only be observed ~0.5% of the time (0.1 * 0.05) if the two binding events were completely independent. However, we observed an order of magnitude greater frequency (~6%) of both proteins being bound. This strongly indicates that forming the SNARE/syt1C2AB/complexin complex is energetically favorable and thus a preferred conformational state. So, either: 1) binding of complexin stabilizes C2AB, 2) binding of C2AB stabilizes complexin, or 3) both proteins stabilize each other when co-bound to a *trans*-SNARE complex.

These new data have been included in a revised version of Figure 7, and the accompanying Results text has been modified:

"The key finding was that many of the individual *trans*-SNARE complexes associated with both syt1-C2AB and complexin (5.3 ± 0.5%; 12 fields of view; 806 total *trans*-SNARE complexes; Fig. 7D), consistent with previously reported ensemble experiments that examined ternary *cis*-SNARE complexes[45]. Moreover, *trans*-SNARE complexes were significantly more likely to harbor both complexin and syt1-C2AB than predicted by chance; under these conditions, the observed frequency of complexin alone (9.8 ± 1.0%) and syt1-C2AB alone (5.7 ± 1.0%) predict a mere 0.6 ± 0.1% chance of randomly observing both molecules bound to the same complex (Fig. 7E). Thus, Ca^{2+}•syt1 and complexin can concurrently bind to the same *trans*-SNARE complex and, interestingly, the concurrent binding of both molecules appears to be energetically favorable (Fig. 7E)."

And from the Figure 7 Legend:

"*D. Left.* Quantification of the frequency that *trans*-SNARE complexes were bound to either complexin alone, C2AB alone, or both. *Right.* Dot plot of the distribution of *trans*-SNARE complexes quantified per experiment. *E*. The random chance of observing both C2AB and complexin bound to the same *trans*-SNARE complex, assuming independence between the binding events, was calculated by multiplying the individual binding frequencies that were observed in each experiment. Interestingly, the observed frequency of the three-protein complex was much higher than predicted by random chance calculation."

p6, Fig 5: These results are very interesting, but are entirely dependent on the relative expression of each of these variants. Do these variants express equally as well as their relative C2A and C2B controls? Are they localised correctly to synaptic vesicles? The lack of effect of some variants (eg the D230,232N mutant) may simply be due to this variant not expressing/ localising to SVs correctly, thus recapitulating the KO phenotype.

We thank the reviewer for pointing out these necessary controls. We first note that only a single mutant (syt1-C2A K189-192A) recapitulated a KO phenotype (the D230,232N displayed elevated mini frequency over KO, similar to syt1-C2A), suggesting that the majority of constructs were likely to have localized.

Newly included data demonstrate that all variants are well-expressed and appear to localize equally as well as the non-mutated truncation constructs. These data have been included in a new Supplemental Figure 4.

In these new ICC experiments, we calculated the Pearson's correlation for the total pool of each syt1 mutant used in Figure 5 - surface and internal were not separated - with synaptophysin. We found no significant difference between the non-mutated syt1-C2A and its three corresponding mutants. Similarly, we did not find a difference between the

non-mutated syt1-C2B and its four corresponding mutants. We interpret these data to indicate that each mutant had the same localization as the non-mutated correlative; i.e. the mutations must not have affected the localization of the domain-deletion construct.

The following changes have been made to the Results text, and a new Supplemental Figure 4 has been added to this manuscript:

"To determine the mechanism by which syt1-C2A increased the frequency of minis (Fig. 2E), we introduced point mutations to selectively disrupt the following key features in this domain: the Ca^{2+}-coordinating ligands (D230,232N)[32,33], membrane-penetrating residues located at the tips of the Ca^{2+}-binding loops (M173, F234A)[14], and the poly-lysine patch (K189-192A)[34,35] (Fig. 5A). None of these mutations alter expression or localization compared to syt1-C2A (Supp. Fig. 4)."

"To gain insight into the molecular mechanisms that enable syt1-C2B to both clamp and synchronize fusion, we conducted structure-function experiments. Analogous to the syt1-C2A experiments above, the Ca^{2+}-coordinating residues (D363,365N) (Earles et al., 2001; Nishiki and Augustine, 2004), the membrane-penetration residues on the Ca^{2+}-binding loops (V304A, I367A) [14], and the poly-lysine patch (K326,327E) [14] in syt1-C2B were disrupted by mutations (Fig. 5D). Additionally, arginine residues 398 and 399, thought to be important for binding t-SNARE heterodimers, were substituted to glutamine (R398,399Q)(Fig. 5D) [38,39]. These mutations did not alter expression or localization compared to syt1-C2B (Supp. Fig. 4). These constructs were then expressed in syt1 KO neurons to determine their impact on the syt1-C2B phenotype."

p11: 'over the course of evolution, the ability to clamp fusion may have migrated from complexin to the C2B domain of syt1'. There is no supporting evidence provided for this speculation. How conserved are, for example, mouse and drosophila synaptotagmin and complexin? How conserved is the C2B domain? Are there different isoforms of complexin and synaptotagmins in mammals vs invertebrates? If anything, other studies suggest that the two proteins interact to clamp spontaneous release in invertebrates (Jorquera + Littleton JT 2012 J Neurosci. Complexin null flies show increased frequency of minis, synaptotagmin null flies are no different from WT, and the complexin-dependent increase in minis is ablated when these flies are crossed with syt null flies).

We thank the referee for this comment. It has been previously postulated that the role of complexin, specifically, has evolved across species. Cross-species rescue experiments, examining complexin null mice and flies, have nicely illustrated this point (the now cited review goes over these experiments and the evidence regarding the evolution of complexin function). So there are differences in the complexin orthologs between mammals and invertebrates and these differences have been examined. In our current study, we are merely extending this line of reasoning: if complexin is no longer a clamp, but syt1 is, then maybe the loss of the complexin clamp, and the gain of syt1 clamping function were coinciding events.

To make it more clear that the function of complexin changed over the course of evolution, and to provide support for this hypothesis, we have modified the results text (shown below). However, as we clearly indicate that this is hypothesis is speculative, we prefer to leave this matter in the discussion.

"We find it intriguing that complexin clearly functions to limit fusion in invertebrate neurons[57,58] and speculate that, over the course of evolution, the ability to clamp fusion may have migrated from complexin to the C2B domain of syt1. Indeed, the function of complexin is hypothesized to have evolved between invertebrates and mammals[15], and the C2B domain of syt1 does not appear to clamp fusion in *Drosophila* neurons[49]."

Minor
p2: 'All studies agree that loss of complexin impairs evoked SV fusion'. Please reference these studies.

This topic was well covered in a recent review [Trimbuch and Rosenmund 2016 *Nat. Rev. Neurosci*]. In the interest of brevity, we have opted to cite this review, along with the new paper from the Brose lab (Cell Reports, 2019) showing that acute disruption of complexin does not unclamp fusion in mammalian central neurons.

p3: The RRP has previously been shown to be not altered in the absence of syt1 alone (Bacaj 2015 PLoS Biology), but only reduced when syt1 and syt7 are both depleted from neurons. These conflicting results should be referenced.

Multiple laboratories using multiple approaches have found that the RRP is significantly reduced in syt1 KO preparations. This has been shown by sucrose measurements, from our lab and many others (Change et al., 2018 *Nat Neuro*; Xue et al., 2015 *PNAS*; Evans et al., 2016 *Mol Bio*; Bai et al., 2016 *Nat Comm*; Evans et al., 2015 *J Neuro*; Liu et al., 2014 *Nat Neuro*; Liu et al., 2009 *J Neuro*; Yoshihara and Littleton, 2002 *Neuron*), as well as by EM visualizing a decreased pool of docked vesicles (Reist et al., 1998 *J Neurosci*; Liu et al., 2009 *J Neurosci*; Change et al., 2018 *Nat Neuro*). The data presented in the current manuscript supports these findings, showing that expression of syt1-FL rescues the RRP in syt1 KO neurons. In contrast, only a single laboratory (Sudhof) has claimed that loss of syt1 does not alter the RRP (Geppert 1994, Sun 2007, Bacaj 2013, Maximov 2005, Xu 2009, and Bacaj 2015). Many of these studies that did not find a difference in RRP utilized autaptic cultures, which fail to reproduce many of the traditional phenotypes associated with syt1 (Liu et al., 2009). Furthermore, other studies from the Sudhof laboratory found that syt1 KO neurons rescued with specific syt1 mutant constructs have a reduced RRP (i.e. Rhee at l., 2005 *PNAS*) (these studies, however, fail to report the RRP of the syt1 KO background) and syt1 KO reduces the RRP of chromaffin cells (Nagy et al., 2006 *J Neuro*). Finally, RRP measurements from the Sudhof laboratory have been previously called into question due to technical concerns (please see Trimbuch and Rosenmund, 2016; bottom of page 120). Given that Bacaj et al. (2015) cannot be reproduced, we prefer to not cite this study, but rather to go with the preponderance of data from all other groups (Brose, Rosenmund, Reist, Littleton, Verhage, Chapman, etc.).

p6 (related to fig 4): 'did not alter the amount of native protein (data not shown)'. Determined how? This result should be included in supplementary data.

This was determined by western blotting – though two particular details of the antibodies against syt1 made this somewhat complicated as follows: 1) the 48.1 antibody recognizes the C2A domain and thus does not label our syt1-C2B construct, and 2) the luminal domain (LD) antibody recognizes all of our constructs (which are the rat variant of syt1) but has a much lower affinity for the endogenous mouse protein. So, to calculate the degree of overexpression in mouse syt1 KO neurons, we had to perform two

separate blots using the same input materials. The first, using 48.1, quantified the expression of syt1-FL and syt1-C2A compared to endogenous levels in WT neurons. The second blot, using the LD antibody, quantified the levels of syt1-C2B relative to syt1-FL and syt1-C2A. From these two blots, we could solve for the relative expression of all three constructs compare to the endogenous syt1 levels in WT neurons. Furthermore, from the 48.1 blot we could determine that the endogenous levels were not altered.

So, while we did the above analysis in mouse neurons to justify the statements in the results, we would prefer to present a simplified example in the manuscript.

We have now included an example blot from WT rat cortical neurons demonstrating: 1) the degree of over expression of each construct, and 2) that the endogenous levels of syt1 were not altered by these viruses. Using rat cortical neurons greatly simplifies the presentation of this point by enabling a single antibody (the luminal domain antibody) to be used for all comparisons. As an aside, electrophysiological experiments conducted in these cultured rat neurons faithfully reproduced the results we found in mouse neurons (Fig. 4). This new blot is presented in Supp. Fig. 3 as panel A.

Methods: details of mutants are missing from methods. Please provide additional details. It is not readily apparent that the mutations in fig 5 are present in the syt1-C2A or syt1-C2B constructs (rather than in full length syt1).

We thank the reviewer for noting the ambiguity in Figure 5 – this has been corrected in the revised manuscript in the legend for Figure 5 (in red):

"*A.* Point mutations were introduced in the syt1-C2A domain-deletion construct. Crystal structure of C2A[33] illustrating the locations of the mutants employed in this study. Ca^{2+} ions (orange) were drawn to illustrate the approximate Ca^{2+} binding sites. *B and C*. Example traces (*B*) and quantification (*C*) of the frequency of mIPSCs in syt1 KO neurons expressing the indicated mutant construct of syt1-C2A. *D.* Point mutations were also introduced in the syt1-C2B domain-deletion construct. NMR structure of C2B [67]; again, the position of each substitution is shown and Ca^{2+} ions (orange) were drawn to illustrate the approximate Ca^{2+} binding sites."

Please provide more details for transfection of neurons for localisation experiments.
Please provide details of what 'n' denotes for Fig 1 (localisation experiment). Are these also from multiple individual cultures?

The following has been included in the Methods section:

In brief, 4 µg of plasmid DNA in 250 mM $CaCl_2$ was added dropwise, with brief vortexing after each drop, to an equal volume of 2x HBS (in mM: 275 NaCl, 10 KCl, 1 Na_2HPO_4, 15 D-glucose, and 40 HEPES pH 7.05). This mixture was incubated for 20 min at RT and then added to the media for one coverslip of cultured neurons. Neurons was then incubated for 45 min at 37°C in 5% CO_2. Afterwards, the media was exchanged for conditioned media pre-equilibrated to 10% CO_2 and maintained at 37°C in a 5% CO_2 humidified incubator.

And separately,

For ICC analysis, an ‘n’ was considered to be a single field of view and n’s were collected from 2-3 separate dissections for each condition.

Reviewers' comments:

Reviewer #1 (Remarks to the Author):

The authors have addressed all my concerns in full. This paper represents a major advance in the field and I strongly support publication in the current form.

Reviewer #2 (Remarks to the Author):

The authors addressed my comments appropriately.

Reviewer #3 (Remarks to the Author):

This remains a very interesting paper, and I believe is worthy of a place in Nature Comms. However, this relies on the authors clearly establishing that the super-clamping ability of syt1-C2B does not simply arise from its plasma membrane localisation.

The authors have addressed the majority of my comments satisfactorily, however there remains the major unaddressed issue of the plasma membrane localisation of the syt1-C2B construct. This is an essential control experiment.

Regarding the author's statement that: “This is notable because over-expressing syt1 in otherwise WT neurons (corresponding to the high expression paradigm in the new supplemental figure) does not result in a super clamped (or any other observed) phenotype”

What level of overexpression are the authors using in the WT cultures? Supp fig 3A would suggest that the authors are achieving extraordinarily high levels of expression/ overexpression (perhaps 5-10x endogenous syt1?) of their lentiviral constructs (although this is from a single western blot, and not quantified), presumably using the 1x dose – though this is unclear. This certainly suggests that in a KO system, that would be lacking the very small endogenous syt1 band (relative to the very highly expressed exogenous band), that this 1x dose would still equate to a very strong overexpression of constructs, rather than a rescue of expression.

The authors have then shown in Supp fig 3B that it is not until they add 10x this dose (to KO neurons? Again this is unclear, but in text of paper they suggest this assay has been performed in KO neurons) that there is a substantial increase in plasma membrane localised syt1-FL (relative to internal syt1-FL).
It is therefore entirely unclear how these dosings relate to the argument that using the 1x dose (rather than the 10x dose) to overexpress syt1-FL in WT neurons in Fig 4 would increase plasma localised syt1-FL. In fact, if anything they add credence to the argument that such a highly overexpressed syt1-C2B construct (as seen in Supp Fig3A, and i.e. would still be highly overexpressed in KO neurons) that is predominantly plasma membrane localised would have the capacity to act as a superclamp simply by being plasma membrane localised.

Therefore, the authors have not yet confirmed that significantly loading the plasma membrane with syt1 does not cause a superclamping phenotype and underlie the ability of syt1-C2B to acts as a superclamp.

This reviewer suggests that the following experiments be performed in order to clarify the results from this paper:

1) Perform the surface pHluorin assay as previously suggested. If the authors find that there is a large

amount of surface stranded syt1-C2B in KO neurons, but there is ALSO an equivalent amount of surface stranded syt-FL when overexpressed in WT neurons (all using the 1x viral dose) then this would confirm the author's assertion that surface stranding of syt1 does not underlie the superclamp phenotype. The authors' statement that "The propensity for syt1 to utilize the PM as a reservoir for over-expressed protein makes the proposed pHluorin experiment quite difficult to perform and interpret" is incorrect, and in fact, would PROVE the very point that they are trying to make (i.e. that surface stranded syt1 doesn't underlie the clamping effect).

OR

2) Perform quantitative immunolabelling of syt1-FL, syt1-C2A and syt1-C2B in WT neurons (using 1x dose), and look at TOTAL synaptic levels of expression (of non-transduced vs transduced synapses in same field of view). Some of the ultra-high levels of overexpression of these constructs in Supp3A might come from extra-synaptic sources. Then, perform the quantitative immunolabelling of syt1-FL, syt1-C2A and syt1-C2B in WT neurons (using 1x dose), and look at SURFACE synaptic levels of expression (of non-transduced vs transduced synapses in same field of view). If indeed using the 1x viral dose in WT neurons results in a significant surface stranding of syt1-FL, which is equivalent to syt1-C2B, this would again add validity to the author's assertion that surface stranding of syt1 does not underlie the superclamp phenotype

Reviewer #3 (Remarks to the Author):

This remains a very interesting paper, and I believe is worthy of a place in Nature Comms. However, this relies on the authors clearly establishing that the super-clamping ability of syt1-C2B does not simply arise from its plasma membrane localisation.

We thank the referee for their positive assessment of our work, and for the time and effort spent evaluating our responses to the concerns that were raised. We now realize that we were unclear regarding the lone lingering point that the referee raises, as to whether clamping is the result of over-expression and cell surface accumulation of the syt1 constructs. We have addressed this issue in a re-revision, and now feel that we have conclusively rule-out this concern, as detailed below.

The authors have addressed the majority of my comments satisfactorily, however there remains the major unaddressed issue of the plasma membrane localisation of the syt1-C2B construct. This is an essential control experiment.

It appears as though syt1-FL and syt1-C2B were internalized to a more-or-less similar degree in our experiments presented in Figure 1. We computed the overall intensity of the internal and surface fraction of syt1 per field of view for each trial. We then used those intensities to make a ratio of internal/surface intensity. While this measurement has little to no meaning in absolute terms (as different antibodies imaged in different channels were used to stain the internal and surface fractions), it does provide information in a relative sense. Importantly, this ratio was fairly similar for the pH-syt1-FL and the pH-syt1-C2B constructs (Surface/Internal: 1.00 vs. 1.25, $p = 0.45$). So, syt1-C2B does not appear to be selectively retained in the PM as compared to the full length protein, at least not to a significant degree. However, the issue of whether or not surface stranded syt1, in general, would clamp fusion is very interesting. This was the prompt for the overexpression experiments included in the initial revision, in which we were able to experimentally increase the surface fraction of syt1 to a large and significant degree. Poor writing, on our part, hindered the interpretation of these experiments; the remainder of this reply is aimed at better explaining these experiments with better language and with the inclusion of new data.

Regarding the author's statement that: "This is notable because over-expressing syt1 in otherwise WT neurons (corresponding to the high expression paradigm in the new supplemental figure) does not result in a super clamped (or any other observed) phenotype"

Because we have worked on syt1 for so long, and know that massive overexpression (from quantitative immunoblots) has no measureable effect, we stated this. However, we also failed to clearly describe and relate the degrees of over-expression in the current manuscript, esp. between the physiology in Fig. 4 and the surface localization in Supp. Fig. 3B, C. We address this with new, quantitative immunoblots, as detailed below.

What level of overexpression are the authors using in the WT cultures? Supp fig 3A

would suggest that the authors are achieving extraordinarily high levels of expression/ overexpression (perhaps 5-10x endogenous syt1?) of their lentiviral constructs (although this is from a single western blot, and not quantified), presumably using the 1x dose – though this is unclear. This certainly suggests that in a KO system, that would be lacking the very small endogenous syt1 band (relative to the very highly expressed exogenous band), that this 1x dose would still equate to a very strong overexpression of constructs, rather than a rescue of expression.

We apologize for the confusion; this was very ambiguous in our manuscript. In short, different viral titers were used to infect syt1 KO neurons in the rescue experiments (Fig. 2) versus WT neurons in the experiments examining over-expression (Fig. 4).

1) In our KO rescue experiments (Fig. 2), syt1 constructs were expressed at 1-2x the endogenous syt1 levels. These new, quantified, expression level data are now included in a revised Supp. Fig. 1C. These data show that our rescue experiments were conducted at the appropriate expression levels, and that the clamping activity of the C2B construct did not require overexpression.

2) In our WT 'overexpression' experiments (Fig. 4C, D), syt1 constructs were indeed expressed at 5-10 times endogenous syt1 levels, as was previously stated in the Results text. Quantification of this degree of overexpression is now shown in revised Supp. Fig. 3A. Importantly, this high level of overexpression had no effect on synaptic transmission.

These issues are clarified in our second revision.

The authors have then shown in Supp fig 3B that it is not until they add 10x this dose (to KO neurons? Again this is unclear, but in text of paper they suggest this assay has been performed in KO neurons) that there is a substantial increase in plasma membrane localised syt1-FL (relative to internal syt1-FL).

This is a misinterpretation of the experiment and data entirely due to ambiguities in our writing - we apologize for creating this confusion. These pHluorin experiments were conducted in KO neurons, and this information has been included in the Figure Legend. More importantly, <u>the "10x" dose of virus in these pHluorin experiments (Supp. Fig. 3B, C) was equivalent to the expression levels that were examined via electrophysiology in Fig. 4C, D.</u>

In these pHluorin experiments, <u>the "1x virus dosage" in Supp. Fig. 3B, C refers to the titer of pH-syt1 virus that resulted in levels of syt1 expression that are very close to the endogenous levels of the native protein</u> (1.15x endogenous levels, to be precise, as now stated in the legend of the revised supplementary figure). We have now changed the axes labels of this Supp. Fig. to make this distinction more apparent. In more general terms, we adjust the baseline titer of all of our syt1 viruses (in this case, with and without a pHluorin tag) so that, at 1x, they all express roughly endogenous protein levels (1-2 fold endogenous levels); this great facilitates comparisons between various viral constructs. The titer of this specific virus (pH-syt1) was determined by immunoblotting; while we can include this blot if necessary, it is slightly complicated as there is some degree of cleavage between the syt1 and pHluorin moiety (which is accounted for in our analysis) when this fusion construct is expressed in neurons. Follow-up studies are currently examining this phenomenon. In the 10x condition, 10 times this viral titer (again, the 1x titer resulted in near endogenous levels) was applied to coverslips.

So, the same titer of viruses (expressing the untagged syt1 constructs) were applied to neurons in the localization experiments (Supp. Fig. 3B, C) and in the functional experiments illustrated by Fig 4 (which, as stated above, resulted in a 5-10 fold overexpression as compared to the endogenous levels determined by immunoblotting). Therefore:

a) The surface expression of syt1 observed in the 1x condition in the pH-syt1 experiments (using KO neurons; Supp. Fig. 3C) is directly relatable to WT neurons that do not overexpress syt1 (Fig. 4, "WT + GFP"), as the 1x condition yield 'endogenous levels' of syt1, and
b) The surface expression of syt1 observed in the 10x condition in the pH-syt1 experiments (Supp. Fig. 3C) is directly relatable to the WT neurons that overexpress syt1 at 5-10 fold endogenous levels (Fig. 4, "WT + syt1-FL") as both were treated with the same titer of virus (10x) and should have the same degree of syt1 overexpression.

Because syt1 was expressed to the same degree in the pHluorin experiments and the functional experiments, we feel confident in stating that overexpression of syt1 causes a significant increase in the surface fraction of the protein without having any phenotype on synaptic transmission; surface stranding, in this case caused by overexpression, does not result in clamping activity.

It is therefore entirely unclear how these dosings relate to the argument that using the 1x dose (rather than the 10x dose) to overexpress syt1-FL in WT neurons in Fig 4 would increase plasma localised syt1-FL. In fact, if anything they add credence to the argument that such a highly overexpressed syt1-C2B construct (as seen in Supp Fig3A, and i.e. would still be highly overexpressed in KO neurons) that is predominantly plasma membrane localised would have the capacity to act as a superclamp simply by being plasma membrane localised.

As outlined above, these two experiments (the 10x condition in Supp. Fig. 3B, C and the "WT + syt1-FL" condition in Fig. 4C, D) overexpress syt1 to the same degree and are thus directly comparable. Again, overexpression of syt1 causes a massive increase in plasma membrane localization without generating a superclamped phenotype.

Therefore, the authors have not yet confirmed that significantly loading the plasma membrane with syt1 does not cause a superclamping phenotype and underlie the ability of syt1-C2B to acts as a superclamp.

Again, we apologize for our ambiguous descriptions of the experiments included in the first round of revision. We believe that these experiments, with proper documentation and explanation, do confirm that significantly loading the plasma membrane with syt1 does not cause a superclamping phenotype.

This reviewer suggests that the following experiments be performed in order to clarify the results from this paper:

1) Perform the surface pHluorin assay as previously suggested. If the authors find that there is a large amount of surface stranded syt1-C2B in KO neurons, but there is ALSO an equivalent amount of surface stranded syt-FL when overexpressed in WT neurons (all using the 1x viral dose) then this would confirm the author's assertion that surface stranding of syt1 does not underlie the superclamp phenotype. The authors' statement

that "The propensity for syt1 to utilize the PM as a reservoir for over-expressed protein makes the proposed pHluorin experiment quite difficult to perform and interpret" is incorrect, and in fact, would PROVE the very point that they are trying to make (i.e. that surface stranded syt1 doesn't underlie the clamping effect).

OR

2) Perform quantitative immunolabelling of syt1-FL, syt1-C2A and syt1-C2B in WT neurons (using 1x dose), and look at TOTAL synaptic levels of expression (of non-transduced vs transduced synapses in same field of view). Some of the ultra-high levels of overexpression of these constructs in Supp3A might come from extra-synaptic sources. Then, perform the quantitative immunolabelling of syt1-FL, syt1-C2A and syt1-C2B in WT neurons (using 1x dose), and look at SURFACE synaptic levels of expression (of non-transduced vs transduced synapses in same field of view). If indeed using the 1x viral dose in WT neurons results in a significant surface stranding of syt1-FL, which is equivalent to syt1-C2B, this would again add validity to the author's assertion that surface stranding of syt1 does not underlie the superclamp phenotype

The work detailed above is in line with the first suggestion from the referee: we believe that we had demonstrated that overexpression of syt1-FL to the same high degree – *it was this key point that we failed to communicate in our original revision* - greatly increases the surface fraction of syt1 (shown via immunolabeling, Supp. Fig. 3B, C) without imparting a clamping phenotype (Fig. 4C, D). In short; surface stranded syt1 doesn't underlie the clamping effect.

Whether "some of the ultra-high levels of overexpression of these constructs in Supp3A might come from extra-synaptic sources" is a very insightful concern. Importantly, the high degree of overexpression in these experiments did not cause an abnormal, non-synaptic distribution of surface syt1. Using the data from Supp. Fig. 3B, C, we have now determined that the Pearson's Correlation between surface-localized syt1 and synaptophysin (our synapse marker) was the same for the high-expression, 10x condition and near-endogenous expression, 1x condition (0.325 vs. 0.35, $p = 0.54$). So, this is not a confounding factor in our experiments.

REVIEWERS' COMMENTS:

Reviewer #3 (Remarks to the Author):

The authors have now addressed all of my queries, and have increased the clarity of the manuscript. I congratulate them on a fine piece of work.

Response to Reviewer Comments.

REVIEWERS' COMMENTS:

Reviewer #3 (Remarks to the Author):

The authors have now addressed all of my queries, and have increased the clarity of the manuscript. I congratulate them on a fine piece of work.

> We thank the referee for their valuable suggestions and are delighted to hear that they have approved of the revised manuscript.
